# Depiction of neuroendocrine features associated with immunotherapy response using a novel one-class predictor in lung adenocarcinoma

**DOI:** 10.1007/s12672-023-00693-4

**Published:** 2023-05-18

**Authors:** Hao Liu, Yan Han, Zhantao Liu, Liping Gao, Tienan Yi, Yuandong Yu, Yu Wang, Ping Qu, Longchao Xiang, Yong Li

**Affiliations:** 1 Department of Oncology, Renmin Hospital, Hubei University of Medicine, Shiyan, 442000 Hubei People’s Republic of China; 2 Department of Oncology, Xiangyang Central Hospital, Affiliated Hospital of Hubei University of Arts and Science, No.136 Jingzhou Street, Xiangyang, 441021 Hubei People’s Republic of China; 3 Department of Gastroenterology, Hubei Clinical Center and Key Lab of Intestinal and Colorectal Diseases, Zhongnan Hospital of Wuhan University, Wuhan, 430072 Hubei People’s Republic of China; 4 Department of Science and Education, Renmin Hospital, Hubei University of Medicine, Shiyan, 442000 Hubei People’s Republic of China; 5 Institute of Cancer Research, Renmin Hospital, Hubei University of Medicine, Shiyan, 442000 Hubei People’s Republic of China

**Keywords:** OCLR, Machine learning, Lung adenocarcinoma, Neuroendocrine differentiation, Immunotherapy

## Abstract

**Background:**

Tumours with no evidence of neuroendocrine transformation histologically but harbouring neuroendocrine features are collectively referred to as non-small cell lung cancer (NSCLC) with neuroendocrine differentiation (NED). Investigating the mechanisms underlying NED is conducive to designing appropriate treatment options for NSCLC patients.

**Methods:**

In the present study, we integrated multiple lung cancer datasets to identify neuroendocrine features using a one-class logistic regression (OCLR) machine learning algorithm trained on small cell lung cancer (SCLC) cells, a pulmonary neuroendocrine cell type, based on the transcriptome of NSCLC and named the NED index (NEDI). Single-sample gene set enrichment analysis, pathway enrichment analysis, ESTIMATE algorithm analysis, and unsupervised subclass mapping (SubMap) were performed to assess the altered pathways and immune characteristics of lung cancer samples with different NEDI values.

**Results:**

We developed and validated a novel one-class predictor based on the expression values of 13,279 mRNAs to quantitatively evaluate neuroendocrine features in NSCLC. We observed that a higher NEDI correlated with better prognosis in patients with LUAD. In addition, we observed that a higher NEDI was significantly associated with reduced immune cell infiltration and immune effector molecule expression. Furthermore, we found that etoposide-based chemotherapy might be more effective in the treatment of LUAD with high NEDI values. Moreover, we noted that tumours with low NEDI values had better responses to immunotherapy than those with high NEDI values.

**Conclusions:**

Our findings improve the understanding of NED and provide a useful strategy for applying NEDI-based risk stratification to guide decision-making in the treatment of LUAD.

**Supplementary Information:**

The online version contains supplementary material available at 10.1007/s12672-023-00693-4.

## Introduction

Lung cancer is one of the leading contributors to cancer incidence and mortality worldwide [1].According to pathological characteristics, there are two major histological subtypes of lung cancer, small cell lung cancer (SCLC), the most common form of neuroendocrine lung cancer, and non-small cell lung cancer (NSCLC), which accounts for approximately 85% of lung cancer cases and mainly comprises lung squamous cell carcinoma (LUSC) and lung adenocarcinoma (LUAD) [2]. EGFR tyrosine kinase inhibitors (EGFR-TKIs) have become the standard first-line therapy for treating patients with advanced NSCLC harbouring EGFR activating mutations. However, acquired resistance to targeted therapies is inevitable [3]. Histological transformation of LUAD into an aggressive neuroendocrine derivative resembling SCLC is one of the mechanisms underlying acquired resistance to EGFR-TKIs [4]. Thus, investigating the mechanisms underlying neuroendocrine differentiation (NED) is crucial for providing more appropriate individualized treatment recommendations for patients with NSCLC. It has been recognized that neuroendocrine features can be demonstrated by immunohistochemistry or ultrastructural pathology in 10–30% of lung cancers with ordinary non-small cell morphology [5]. These tumours with no histological evidence of neuroendocrine transformation are collectively referred to as non-small cell carcinomas with NED (NSCLC-NED). Some studies have reported that NSCLC-NED was associated with an increased response to chemotherapy, whereas others have not [6, 7].

Immunohistochemistry (IHC) is the main method used to identify NED [8]. The different sensitivities and specificities of neuroendocrine markers, such as chromogranin A (CgA), neural cell adhesion molecule 1 (CD56), synaptophysin (Syp) and neuron-specific enolase (NSE), may lead to significant variation in reported NSCLC-NED incidence and contradictory results about the prognosis of NSCLC-NED [9]. Transcriptomic technologies, especially RNA sequencing, with advantages of being amenable to standardization and avoiding interpretation bias, are more accurate for diagnosing cancer and provide a reliable estimation of protein marker levels in cancer [10, 11]. Hence, integrative analysis based on the transcriptome may enable quantitative assessment of the degree of NED in NSCLC.

Gene expression data obtained from tumour biopsy samples reflect a superposition of cell subpopulations in samples. The methods developed to deconvolute gene expression data from mixed samples are based on nonnegative matrix factorization or other matrix decomposition techniques, and it is difficult to identify all tumour subtypes in a single optimization [12]. The one-class logistic regression (OCLR) machine learning algorithm is a novel one-class method based on logistic regression, which has been demonstrated to be better than its binary predictor counterparts in identifying specific cell types in heterogeneous cell populations [13]. The OCLR algorithm was applied to obtain independent stemness indices in multiple malignancies and revealed patterns of intratumor molecular heterogeneity [14]. It is worth conducting OCLR in the assessment of NED in NSCLC.

Immunotherapy has become a promising tool for cancer treatment, but its efficacy remains limited in most clinical settings [15]. The link between immunotherapy response and neuroendocrine features of NSCLC is unclear. There is still a lack of reliable biomarkers for predicting immunotherapy benefit in NSCLC. In the present study, we used OCLR to build a new model to quantitatively determine the tendency of NSCLC tumours to undergo NED based on mRNA expression data and named it the NED index (NEDI). This study aimed to develop a classification tool and machine-learning algorithm to analyse a spectrum of neuroendocrine features, predict the efficacy of immunotherapy, and guide decision-making regarding the treatment of NSCLC. Our findings may provide novel insights for improving therapeutic efficacy in NSCLC, especially LUAD.

## Materials and methods

### Acquisition of lung cancer cohorts and data processing

The present study collected lung cancer data from diverse cohorts from four individual platforms: The Cancer Genome Atlas (TCGA), Clinical Proteomic Tumor Analysis Consortium (CPTAC), Cancer Cell Line Encyclopedia (CCLE) and Gene Expression Omnibus (GEO). We downloaded the preprocessed mRNA expression matrices of 185 lung cancer cell lines (50 SCLC cell lines and 135 NSCLC cell lines) from the CCLE data portal (http://www.broadinstitute.org/ccle) [16] to establish a neuroendocrine features model by applying the OCLR algorithm. The mRNA expression profiles, clinical information, proteomic data and somatic mutation data of all TCGA-LUAD cohorts and TCGA-LUSC cohorts were obtained through the TCGA data portal (https://portal.gdc.cancer.gov/). The expression profile and clinical information of LUAD and LUSC from CPTAC [17, 18] and SCLC data from the U Cologne SCLC cohort [19] were downloaded from cBioPortal (https:// www. cbioportal.org). In addition, we retrieved and obtained lung cancer datasets from the GEO database, including the GSE159857 cohort [20], GSE60644 cohort [21], GSE9074 cohort (Kano M et al*.*), GSE118131 cohort [22],GSE64322 cohort [23], GSE1307 cohort [24], GSE31625 [25], GSE31210 cohort [26], GSE50081 cohort [27], GSE72094 cohort [28], GSE91061 cohort [29], GSE13213 cohort [30], GSE126044 cohort [31], GSE42127 cohort [32] and GSE135222 [33]. Detailed information about the public datasets used in this study were shown in Table S1. To reduce the systematic error in the different datasets, the gene expression TPM values of RNA-seq were performed log2 transformation using a pseudo-count of 1; log2(TPM + 1) and all microarray data included in this study were log2 transformed and then normalized to [0,1]. The normalization formula was X' = (X-Xmin)/(Xmax-Xmin), where X is the processed microarray value of gene expression [34].

### Cell lines and Western blot analysis

Human lung adenocarcinoma cell lines A549, HCC827 and PC9 were purchased from China Center for Type Culture Collection (CCTCC, Wuhan, China) and human small cell lung cancer cell line H524 was purchased from Procell (Procell, Wuhan, China). HCC827, PC9 and H524 were cultured in RPMI 1640 medium (Gibco, USA) with 10% fetal bovine serum (FBS), 100 U/ml penicillin and 100 U/ml streptomycin. A549 was cultured in DMEM medium (Gibco, USA) with 10% fetal bovine serum (FBS) and penicillin–streptomycin. The whole cell lysates were derived from RIPA lyzed cells and the protein concentrations were determined by the BCA kit (Beyotime). Then, the protein was run on an SDS-PAGE gel and transferred to nitrocellulose. Nitrocellulose membranes were blocked in 5% bovine serum albumin (BSA) and probed with antibodies overnight. Anti-GAPDH, CD56, and Syp were purchased from Cell Signaling Technology; Anti- CgA were purchased from Proteintech. Secondary antibodies conjugated to horseradish peroxidase were followed by enhanced chemiluminescence (Thermo Fisher Scientific, Waltham, Massachusetts, USA).

### Calculation of the NED index (NEDI) for NSCLC

To calculate a NEDI based on mRNA expression, we built a novel model using OCLR trained on lung neuroendocrine cancer from the CCLE data portal using R package “gelnet” [13]. To ensure compatibility between the different cohorts, we first mapped the list of common genes at the intersection of three cohorts (CCLE, TCGA-LUAD and CPTAC-LUAD), and the resulting matrix containing 13,279 mRNAs expression values (log2(TPM + 1)) across all available samples of SCLC and NSCLC cell lines from the CCLE data portal was further analysed. We mean-centred the mRNA expression data and then applied OCLR to only the SCLC samples. When the signature was obtained, it could be applied to score other samples. For mRNA expression data, we calculated Spearman correlations between the model’s weight vector (Table S2) and the new sample’s expression profile (such as NSCLC samples from TCGA or GEO datasets). Then, the Spearman correlations were subsequently mapped to the [0,1] range by using a linear transformation that subtracted the minimum and divided by the maximum and were defined as NEDI.

### Evaluation of the NEDI with somatic variants in TCGA-LUAD

In this analysis, we aimed to investigate the differences in somatic mutations between the NEDI-low and high subgroups according to the median NEDI and further detected the SNVs, SNPs, and INDELs using R software. We downloaded and prepared somatic variants with the Mutation Annotation Format (MAF) and used the R package “maftools” to perform the visualization [35]. The tumour mutation burden (TMB) referred to number of the somatic genes, base substitutions, insertions or deletions, and we calculated the TMB as (mutation frequency with the number of variants)/the length of exons (38 million) for each TCGA-LUAD sample. The associations between NEDI and TMB data were calculated based on Pearson correlation coefficients. Co-occurring and mutually exclusive mutations were identified using the R package “CoMEt” [36]. Fisher’s exact test was used to identify the differential mutation pattern, and genes with a *P* value lower than 0.05 were defined as differentially mutated genes.

### TME construction and immunotherapy response prediction

Immune-related gene signatures were calculated using the single-sample gene-set enrichment analysis (ssGSEA) algorithm through R package “GSVA” [37]. The annotated gene set file (msigdb.v6.2.symbols.gmt) was adopted for our analysis as the reference, and the significance was also based on the threshold of *P* < 0.05. The correlations between the NEDI and all 29 immune-related gene signatures were calculated based on the Pearson correlation coefficients. The stromal, immune, and estimate scores of each sample were estimated using the ESTIMATE algorithm by the R package “estimate”[38]. GSEA was performed by the R package “fgsea”. The Cancer Immunome Atlas (TCIA) web tool provides the results of comprehensive immunogenomic analyses (https://tcia.at/) [39]. Tumour immunogenicity was quantitatively scored from 0 to 10 and was named the immunophenoscore (IPS). Next, we also used the ssGSEA algorithm to compare the T-cell inflamed gene expression profile (GEP) and innate anti-PD-1 resistance (IPRES), which could predict melanoma response to ICB treatment between the NEDI-low and high subgroups. Furthermore, the unsupervised subclass mapping (Submap) method was applied to evaluate the expression similarity of LUAD patients in the NEDI-low and high subgroups with different immunotherapy outcomes [40]. If a pair's expression profiles shared more similarity, their clinical outcomes were more likely to be similar [41].

### Assessment of sensitivity to chemotherapeutic and molecular drugs

To estimate the NEDI in predicting the response to chemotherapeutic and molecular drugs, the R package “pRRophetic” [42] was applied to calculate the half-maximal inhibitory concentration (IC50) of samples between the NEDI-low and high subgroups. The IC50 values of the NEDI-low and high subgroups were compared with the Mann–Whitney test.

### Screening of potential compounds targeting the NEDI signature in LUAD

The Connectivity Map (CMap) [43] dataset is a comprehensive resource for exploring the underlying associations among genes, chemicals, and biological conditions. We wanted to utilize the CMap to screen potential candidate compounds that target crosstalk associated with LUAD in the NEDI-low and high subgroups. We calculated a list of differentially expressed genes between NEDI-low and high subgroups and selected the top 150 to query the dataset. The query results were calculated and ranked with scores from -100 to 100. Overall, we selected the relative compounds that had an enrichment score ≥ 70, which might be capable of targeting the NSCLC-NED.

### Establishing and evaluating the prognosis prediction model

Survival time and status were used to evaluate the prognosis of LUAD patients. The workflow to explore the prognostic signatures in LUAD consists of four steps: (1) Genes were sorted based on a median absolute deviation (MAD) value > 0.5 and a gene co-expression network was constructed using the R package “WGCNA” [44] to analyse and identify gene modules strongly associated with the NEDI in TCGA-LUAD. The 2653 ranked genes were screened and used for further analysis. (2) Univariate Cox proportional hazards regression was used to assess the individual effect of every alteration using the R package “survival”, and then the features with a *P* value less than 0.05 were selected for further analysis. (3) Lasso regression model was used to obtain the genetic variable-based prediction model and to generate the risk scores for all samples using the R package “survival”. (4) The risk score of each patient was predicted based on the training model by using the “predict” function included in the R package “survival”. In our analysis, 9 genes (CXCL17, DKK1, KRT8, IRX5, ENPP5, RHOV, MELTF, ESYT3, PLEK2) retained their Lasso regression coefficients. LUAD cohorts from GEO (GSE72094, GSE31210 and GSE50081) were used as validation sets. Based on the gene expression values and Lasso coefficients, a risk score for each sample was calculated as follows:

$$\mathrm{risk\, score}={\sum }_{1}^{n}expression\times coefficient$$

Then, we conducted survival analyses of LUAD patients with respect to risk level. To further improve the prediction ability, we constructed a nomogram by integrating stage and risk score by R package “survival” and “rms” [45]. Time-dependent receiver operating characteristic (time-ROC) analysis and time-dependent concordance index (C-index) were used to validate the best accuracy of nomogram compared with other prognostic factors by R package “pec” and “riskRegression” [46, 47]. The time-ROC and C-index were calculated to evaluate the performance of the prognosis prediction model.

### Statistical analysis

Student’s t test was used to compare two groups with normally distributed data, while the Mann–Whitney test was conducted to compare groups with nonnormally distributed data. For paired samples, the paired t-test was utilized. Comparisons between multiple groups with normally distributed data were carried out using an analysis of variance (ANOVA). Differential analysis was performed based on the R package “limma” [48], and Kaplan–Meier analysis was conducted via the R package “survival” by using the optimal cut-off points. Analyses were conducted using GraphPad V.9.4.1 software and R V.4.2.1 software, and statistical significance was defined as* P* < 0.05. The information about key software and algorithms used in this study are provided in Table S3.

## Results

### Development and validation of the mRNA expression-based one-class predictor to quantitatively evaluate neuroendocrine features in NSCLC

**Fig. 1:**
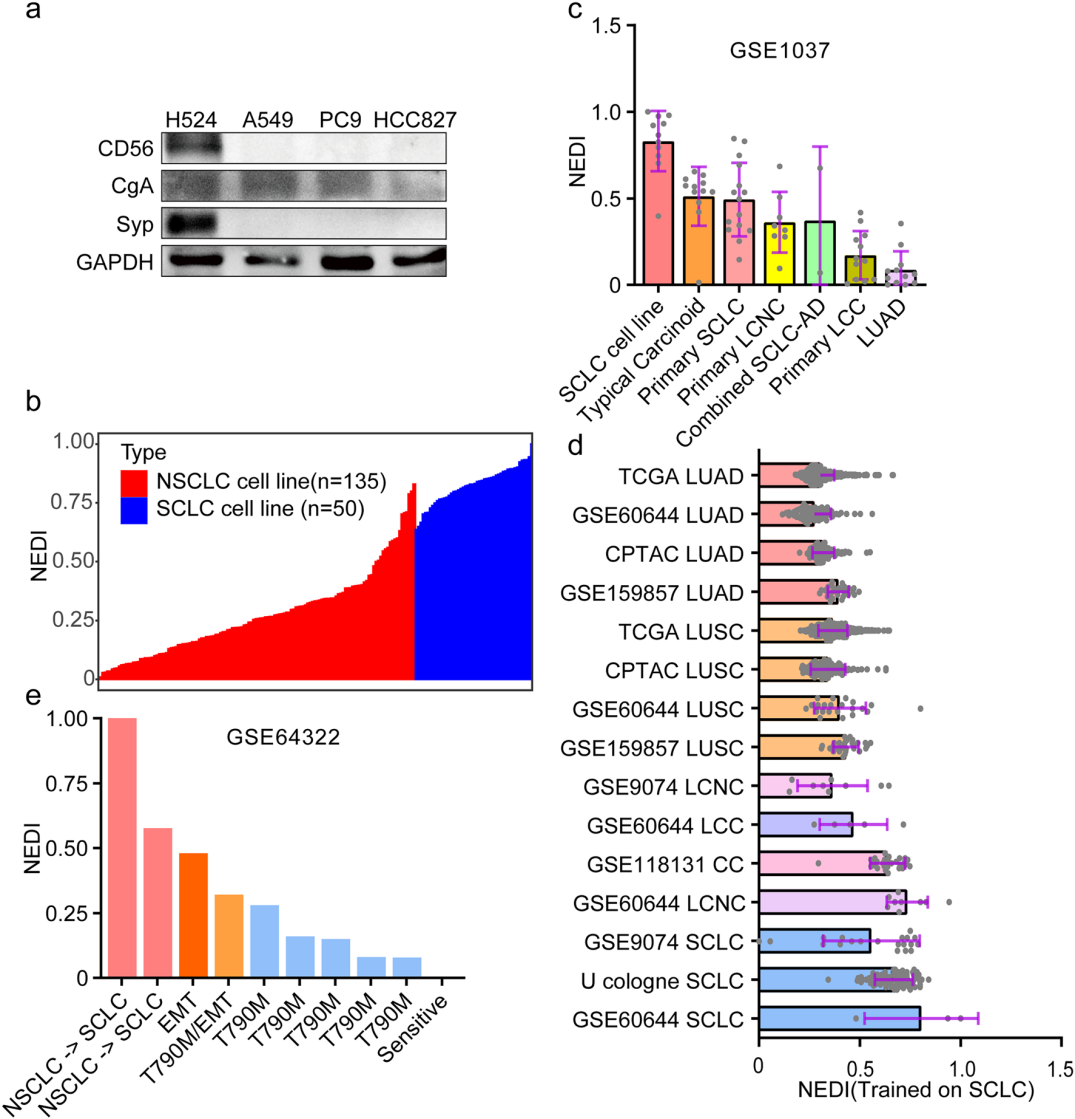
Development and Validation of the NED Index (NEDI). **a** Cell lysates of A549, HCC827 and PC9 human lung adenocarcinoma cells and H524 human small cell lung cancer cells were collected, and neuroendocrine markers including CgA, CD56, and Syp were analyzed by immunoblotting. H524 cells were used as positive control. **b** The NEDI of 185 lung cancer cell lines (50 SCLC cell lines and 135 NSCLC cell lines) from the CCLE data portal were calculated using our model. **c** The NEDI of external dataset (GSE1037) composed of both neuroendocrine cancers, including large-cell neuroendocrine carcinoma, typical carcinoid and SCLC, and non-neuroendocrine lung cancers were calculated. **d** The NEDI of different lung cancer cohorts with distinct histological types were determined. **e** The NEDI of NSCLC samples that are characterized by different molecular changes associated with TKI resistance (GSE64322) were scored

To define the signatures of NSCLC tumours with neuroendocrine features, we first determined the protein levels of neuroendocrine markers, including CgA, CD56, and Syp, in NSCLC cells, and we found that these markers were undetected in A549, HCC827 and PC9 human lung adenocarcinoma cells, except for CgA (Fig. 1a). This result indicated that it is difficult to evaluate neuroendocrine features at the neuroendocrine marker protein level. Hence, we performed an integrative analysis based on the transcriptome to quantify the degrees of NED in lung cancer. Public lung cancer datasets for which transcriptomic profiles were available, including TCGA, CPTAC, CCLE and GEO, were analysed. For the mRNA expression-based model, to ensure compatibility between the different cohorts, we first mapped the list of common genes at the intersection of three cohorts, including CCLE, TCGA-LUAD and CPTAC-LUAD, and the resulting matrix containing 13,279 mRNAs expression values across all available samples was further analysed (Figure S1a). Traditional supervised approaches deconvolute gene expression data from mixed samples into a set of distinct cell types based on two or more classes to train models. However, there is no definitive negative class, and it is unclear how the classes in the negative set should be weighted for neuroendocrine features in NSCLC. Therefore, we developed a NED index (NEDI) using the OCLR algorithm trained on 50 SCLC cells (gene expression datasets from CCLE database), a particular known pulmonary neuroendocrine cell type that exhibits neuroendocrine features, to identify whether neuroendocrine features are present at some appreciable level in an NSCLC tumour sample that may contain numerous cell types (Fig. 1b). The OCLR-based NEDI ranges from low (zero) to high (one) NED and was applied to multiple lung cancer cohorts. The NEDI was validated by applying it to an external dataset composed of both neuroendocrine cancers, including large-cell neuroendocrine carcinoma, typical carcinoid and SCLC, and non-neuroendocrine lung cancers (GSE1037) [24, 49]. As anticipated, lung cancer samples showed different degrees of neuroendocrine phenotypes, and LUAD samples displayed lower NEDI values than neuroendocrine tumour types (Fig. 1c). Within these, individual tumour samples showed heterogeneous neuroendocrine features. Moreover, the NEDI values in different lung cancer cohorts with distinct histological types were further calculated, and all neuroendocrine samples exhibited higher NEDI values than samples from LUAD and LUSC (Fig. 1d). Interestingly, primary tumour LUAD samples exhibited neuroendocrine features that were similar to those of non-tumour samples obtained from matched adjacent normal tissues of origin (Figure S1b). Moreover, we found that the neuroendocrine index using the OCLR algorithm trained on large-cell neuroendocrine carcinomas (LCNC) or carcinoids (CC) could not exactly distinguish neuroendocrine and non-neuroendocrine carcinomas (Figure S1c, d).

Neuroendocrine transformation is one of the mechanisms of acquired resistance to EGFR-TKIs in LUAD. Therefore, we further analysed the NEDI by scoring index values of NSCLC samples that are characterized by different molecular changes associated with TKI resistance (GSE64322), and we found that TKI-resistant NSCLC samples attained a higher NEDI than TKI-sensitive NSCLC samples (Fig. 1e). A similar result was also observed in LUAD with resistance to erlotinib (GSE31625) (Figure S1e). These results suggested that NSCLC samples exhibited NED during TKI resistance even in the absence of detectable histological transformation into SCLC. In addition, we noted that a high NEDI was associated with higher gene expression of neuroendocrine markers, including CgA, CD56, Syp and NSE in TCGA-LUAD and TCGA-LUSC (Figure S1f-m).

In summary, we developed and validated an mRNA expression-based one-class predictor trained on SCLC cells to quantitatively evaluate neuroendocrine features in NSCLC, and lung cancer samples stratified by the index were used for further integrative analyses.

### Selected molecular and clinical outcomes associated with the NEDI in LUAD

**Fig. 2:**
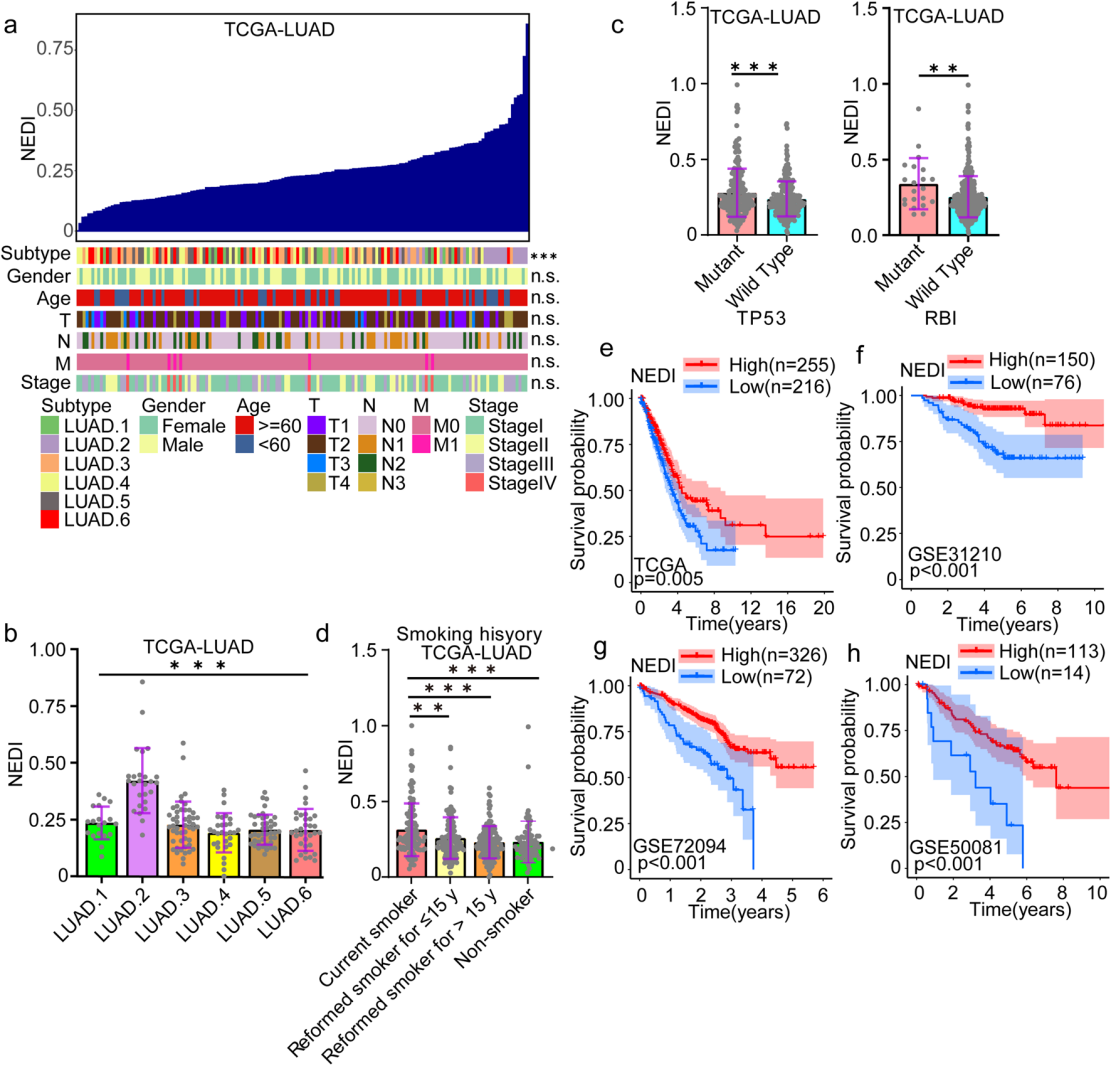
Selected molecular and clinical features associated with the NEDI in LUAD. **a** An overview of the associations between well-known molecular and clinical signatures and the NEDI in TCGA-LUAD. **b** The association of LUAD molecular subtypes with the NEDI in TCGA-LUAD. **c** Comparisons of NEDI values between TP53-mutant and TP53-wild type samples as well as RB1-mutant and RB1-wild type samples in the TCGA-LUAD cohort. Data were analysed by Student's t test. **d** The association of smoking history with the NEDI in TCGA-LUAD. **e**–**h** Kaplan‒Meier curves of overall survival (OS) according to the NEDI values of LUAD patients in the LUAD cohorts. *P < 0.05, **P < 0.01, *** *P* < 0.001

We further investigated the association between the NEDI and the known clinical and molecular features of LUAD. We observed no significant association between the NEDI and clinical features, including gender, age, TNM classification and clinical stage (Fig. 2a). Interestingly, we found that the NEDI was highest in LUAD.2 (Fig. 2b), one of the molecular subtypes of LUAD previously defined in TCGA and known to exhibit higher tumour ploidy and mutation rates and to frequently harbour TP53 mutations [50]. Biallelic inactivation of TP53 and RB1 has been reported to be associated with several key oncogenic processes in SCLC [19]. We found that a high NEDI was strongly associated with somatic mutations in the TP53 and RB1 genes, which are potent tumour suppressors in LUAD (Fig. 2c). This result indicated that TP53 and RB1 could be drivers of the dedifferentiation of neuroendocrine in LUAD. EGFR mutation is a well-known driver in lung cancer [51]. However, we did not find a correlation between the NEDI and EGFR mutation status (Figure S2a-c). These results suggested that aberrant pathway alterations mediated by genetic disorder play an important role in regulating the NED of LUAD and remain to be further investigated.

SCLC is strongly associated with exposure to tobacco carcinogens [52]. Therefore, we evaluated the association between the NEDI and smoking status, and we found that current smokers had a higher NEDI than former smokers and non-smokers (Fig. 2d). This result suggested that the NED of LUAD might be activated in response to smoking. It has been shown that NED is related to the variable prognosis of NSCLC in different studies, with sometimes conflicting results [53]. However, we observed that a higher NEDI correlated with better prognosis in the TCGA-LUAD cohort in the present study (Fig. 2e). Similar results were obtained in other GEO-LUAD cohorts (GSE31210, GSE72094 and GSE50081) (Fig. 2f–h). These findings indicated that the NEDI based on transcriptomic data has a better prognostic value for LUAD than IHC.

### Correlation of the Mutational Landscape and NED in LUAD

**Fig. 3:**
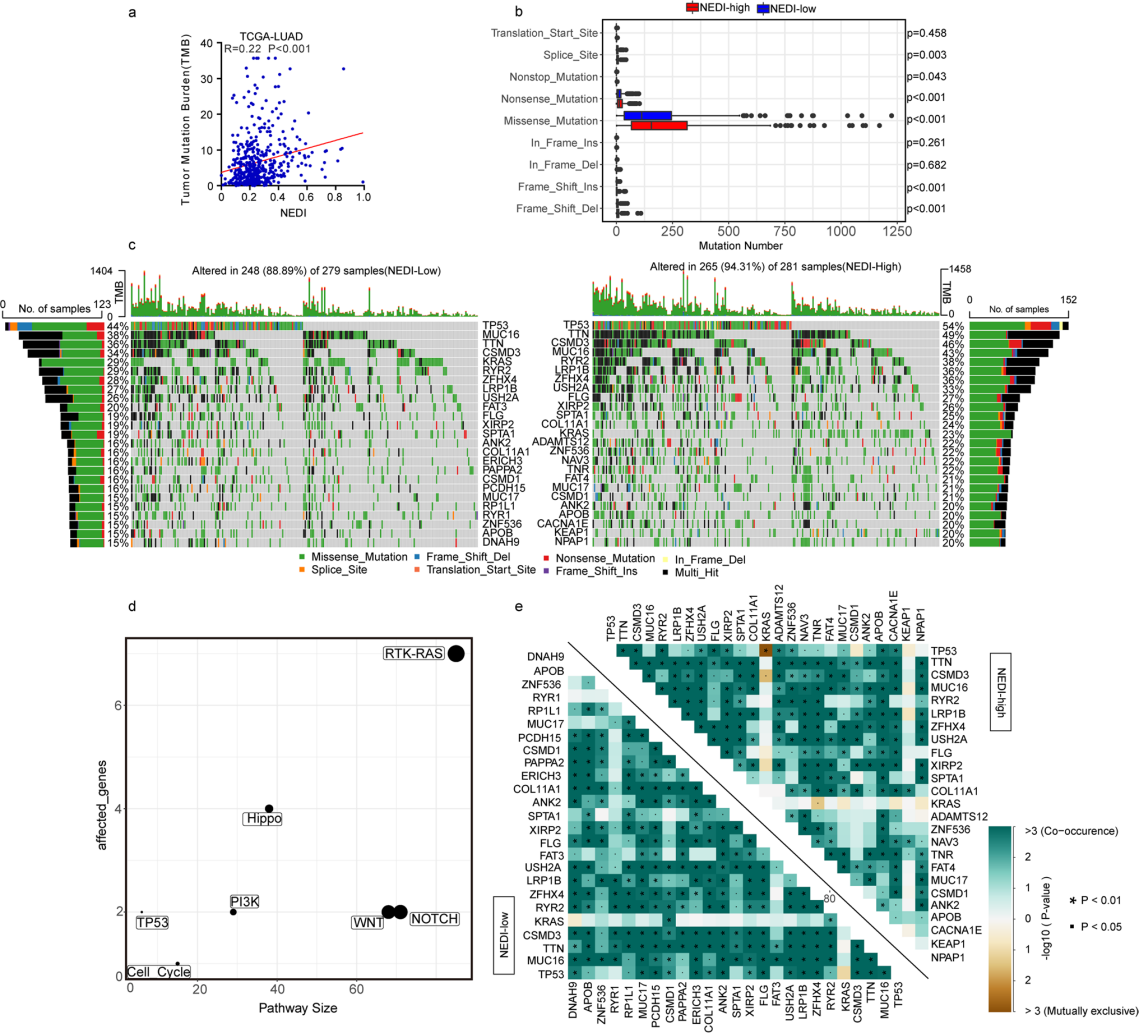
Landscape of somatic mutations in LUAD in Low and High NEDI Subgroups. **a** Correlation analysis between the NEDI and tumour mutation burden (TMB) in TCGA-LUAD. Data were analysed by Pearson correlation analysis. **b** Comparisons of mutation frequencies of every mutation type classified by effects between the NEDI-low high subgroups. **c** The waterfall plot shows the mutation distribution of the top 25 most frequently mutated genes. The central panel shows the types of mutations in each LUAD sample. The upper panel shows the mutation frequency of each LUAD sample. The bar plots on the left and right sides show the frequency and mutation type of genes mutated in the NEDI-low and high subgroups, respectively. **d** Oncogenic pathway analysis showing frequent genetic alterations relative to NED in TCGA-LUAD. **e** The heatmap illustrates the mutually cooccurring and exclusive mutations of the top 25 frequently mutated genes. The colour and symbol in each cell represent the statistical significance of the exclusivity or cooccurrence for each pair of genes

As previously mentioned, genetic disorders might play an important role in regulating NED in LUAD. Therefore, we systematically further investigated the genomic disparity between the NEDI-low and high subgroups, which were divided according to the median NEDI value, in LUAD based on WES data from the TCGA-LUAD cohort. Somatic mutations, including single-nucleotide polymorphisms (SNPs), single-nucleotide variants (SNVs), insertions (INSs), and deletions (DELs), were analysed with the R package “maftools”. As expected, we found a strong association between the NEDI and a higher tumour mutation burden (TMB) (Fig. 3a). We noted that the genomic variants were highly enriched in missense mutations in both the NEDI-low and high subgroups (approximately 85%), and samples in the NEDI-high subgroup exhibited a significantly larger number of variants than those in the NEDI-low subgroup (Fig. 3b). For SNVs, C > A was the most common type of variant, and all SNVs were enriched in tumour samples with a high NEDI (Figure S3a). In addition, we found that a high NEDI was associated with transversion (Tv) in all SNVs; however, an adverse association between the NEDI and transition (Ti) in all SNVs was detected (Figure S3b). We also found that SNPs were enriched in the NEDI-high subgroup (Figure S3c). Despite the significant differences in the variant number of the somatic mutations between the NEDI-low and high subgroups, the constituent ratio of each mutation type occupied among all variants remained almost unchanged (Figure S3d-g), indicating that genomic disparity in variant number was not caused by a shift in the type of mutation.

Moreover, we found that 25 genes were mutated in more than 20% of the samples in the NEDI-high subgroup, while only 10 genes met this criterion in the NEDI-low subgroup (Fig. 3c). Interestingly, TP53, TTN, CSMD3 and MUC16 occupied the top four positions in both subgroups. In addition, 578 differentially mutated genes were detected using Fisher’s exact test (Table S4). We further performed an enrichment analysis of known oncogenic pathways based on these differentially mutated genes and found that 7 canonical pathways, the RTK-RAS, Notch, β-catenin/Wnt, Hippo, PI-3-Kinase/Akt, cell cycle and TP53 signalling pathways, were significantly enriched (Fig. 3d). This suggested that these oncogenic pathway alterations mediated by genomic mutation, especially RTK-RAS signalling pathway alterations, might be related to the regulation of NED in LUAD.

Moreover, we investigated the co-occurring and exclusive mutations of the top 25 most frequently mutated genes by using the CoMEt algorithm. Compared with the pervasive co-occurrence landscape, there were three unique cases (KRAS-TP53, KRAS-CSMD3 and KRAS-TNR) that exhibited mutually exclusive mutations in the NEDI-high subgroup (Fig. 3e), suggesting that these pairs of genes shared the same pathways and probably had redundant effects on regulating the NED of LUAD.

Taken together, these results suggested that the mutations played a meaningful role in the NED of LUAD.

### Biological processes associated with NED in LUAD

**Fig. 4:**
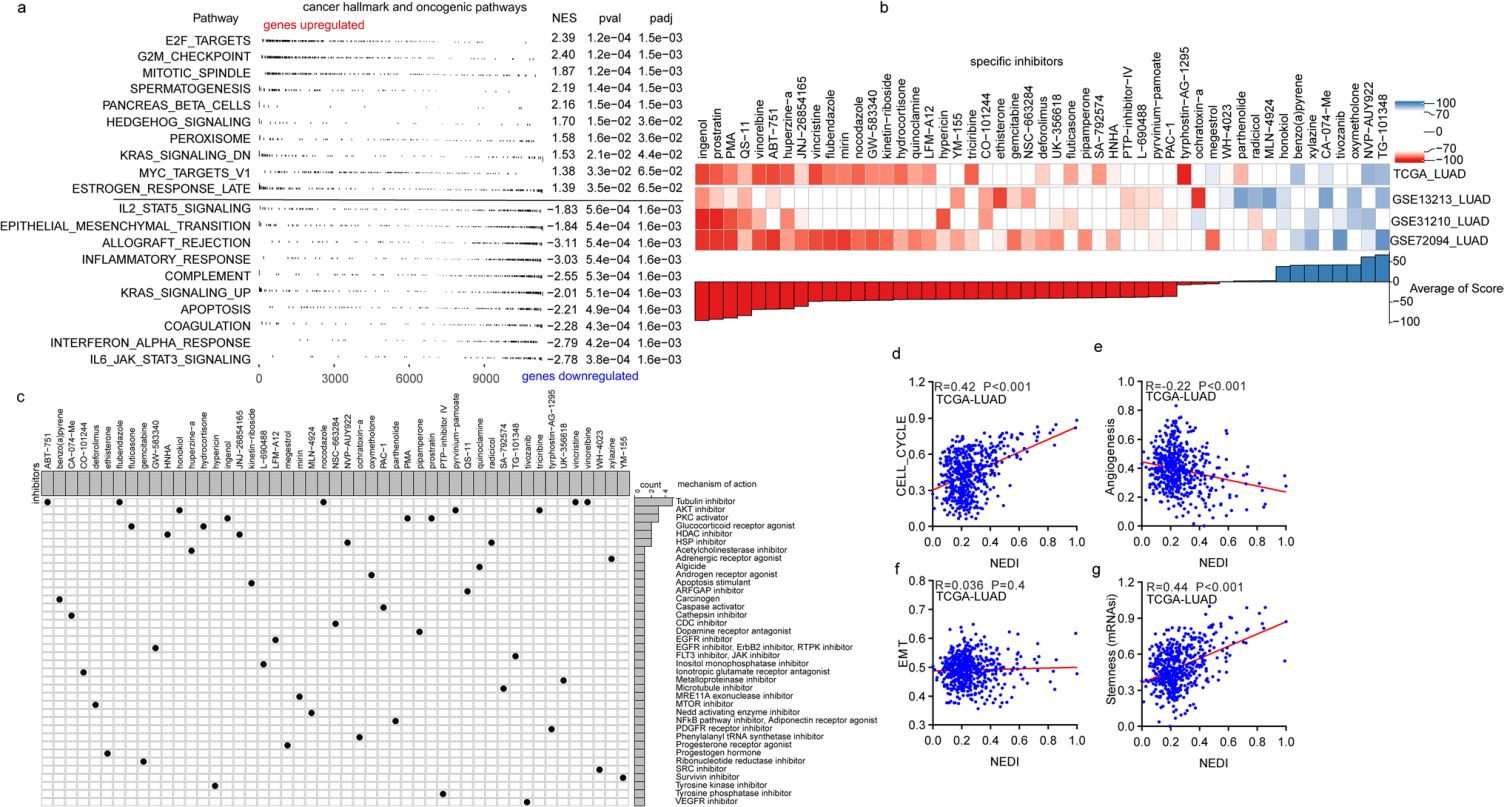
Biological processes associated with the NEDI in LUAD. **a** Gene set enrichment analysis (GSEA) demonstrating the NEDI evaluated in the context of cell-cycle-related cancer hallmark gene sets. NES: normalized enrichment score; padj: adjusted *P* value. **b** Heatmap showing the enrichment score (positive in blue, negative in red) of each compound from the CMap for four LUAD cohorts. Compounds are sorted from right to left by descending number of cohorts significantly enriched. **c** Heatmap showing each compound (perturbagen) from the CMap that shares mechanisms of action (rows) and sorted by descending number of compounds with shared mechanisms of action. **d**–**g** The correlation between NEDI and the malignant features including cell cycle, angiogenesis, EMT quantified by the ssGSEA algorithm and Stemness index (mRNAsi) in TCGA-LUAD. Data were analysed by Pearson correlation analysis

To investigate the biological processes associated with NED in LUAD, pathway enrichment analysis (gene set enrichment analysis, GSEA) was performed on differentially expressed genes (DEGs) between the NEDI-low and high subgroups in the TCGA-LUAD cohort. Notably, cell-cycle-related cancer hallmark gene sets, such as E2F_TARGETs, G2M_CHECKPOINT and MITOTIC_SPINDLE, significantly contributed to the positive side of the NED signature (Fig. 4a). This suggests that the progression of the cell cycle orchestrated by numerous checks and balances might be distinctly primed for NED. However, immune-related cancer hallmark gene sets, such as IL6_JAK_STAT3_SIGNALING, INTERFERON_ ALPHA_RESPONSE and INFLAMMATORY_RESPONSE, were significantly enriched on the negative side of the NED signature (Fig. 4a). This negative enrichment implied that sufficient immune cell infiltration might be exhibited within tumour samples with low NED, and those patients might benefit from immunotherapy.

To further delineate potential molecular events underlying NED in LUAD, the top 150 upregulated and downregulated genes in the NEDI-high subgroup compared with the NEDI-low subgroup in the TCGA-LUAD and 3 GEO-LUAD cohorts were subjected to the CMap (http://www.broad.mit.edu/cmap/) analysis, a systematic approach to investigate the functional connections among diseases, genetic perturbations, and drug actions. CMap mode-of-action (MoA) analysis revealed 38 mechanisms of action shared by 49 compounds with the highest prediction scores. In particular, 5 compounds (ABT-751, flubendazole, nocodazole, vincristine, vinorelbine) shared the MoA of tubulin inhibitors (Fig. 4b, c), suggesting that microtubule-based functions, such as cell migration, cell division, and intracellular trafficking, might be involved in NED and that microtubule-targeting agents, such as taxanes (paclitaxel and docetaxel) and vinca alkaloids (vincristine), might mediate the dedifferentiation of neuroendocrine in LUAD.

To investigate the association between the NEDI and these malignant features, including rapid proliferation [54], angiogenesis [55] and active EMT(obtained from Gene Ontology (GO): 0001837), we quantified the differentiation ability of tumours in the cell cycle, angiogenesis and EMT by the ssGSEA algorithm and found pronounced positive correlations of the NEDI with the cell cycle and negative correlations of the NEDI with angiogenesis in TCGA-LUAD and GEO-LUAD cohorts(GSE13213, GSE50081 and GSE72094) (Fig. 4d, e and Figure S4a,b). However, no significant association of the NEDI with EMT was observed (Fig. 4f and Figure S4c). It has been reported that cancer progression involves a gradual loss of a differentiated phenotype and an acquisition of stem-cell-like features [14]. Therefore, we also tested the association of the NEDI with the stemness index. We observed a positive correlation between the NEDI and the stemness index (Fig. 4g and Figure S4d), suggesting that LUAD samples with high levels of neuroendocrine features exhibited progenitor and stem-cell-like features, which might display the potential for histological transformation into SCLC. These results indicated that with more active NED, there was more rapid cell proliferation and reduced angiogenesis within microenvironments in LUAD and that LUAD samples with a high NEDI might be more susceptible to chemotherapy and less sensitive to antiangiogenic agents.

In conclusion, microtubule-based functions, such as cell migration, cell division, and intracellular trafficking, might be involved in NED, and tumours with strong potential for NED were generally accompanied by more rapid cell proliferation and reduced angiogenesis within microenvironments in LUAD.

### Association of the NED index with immunotherapy response in NSCLC

**Fig. 5:**
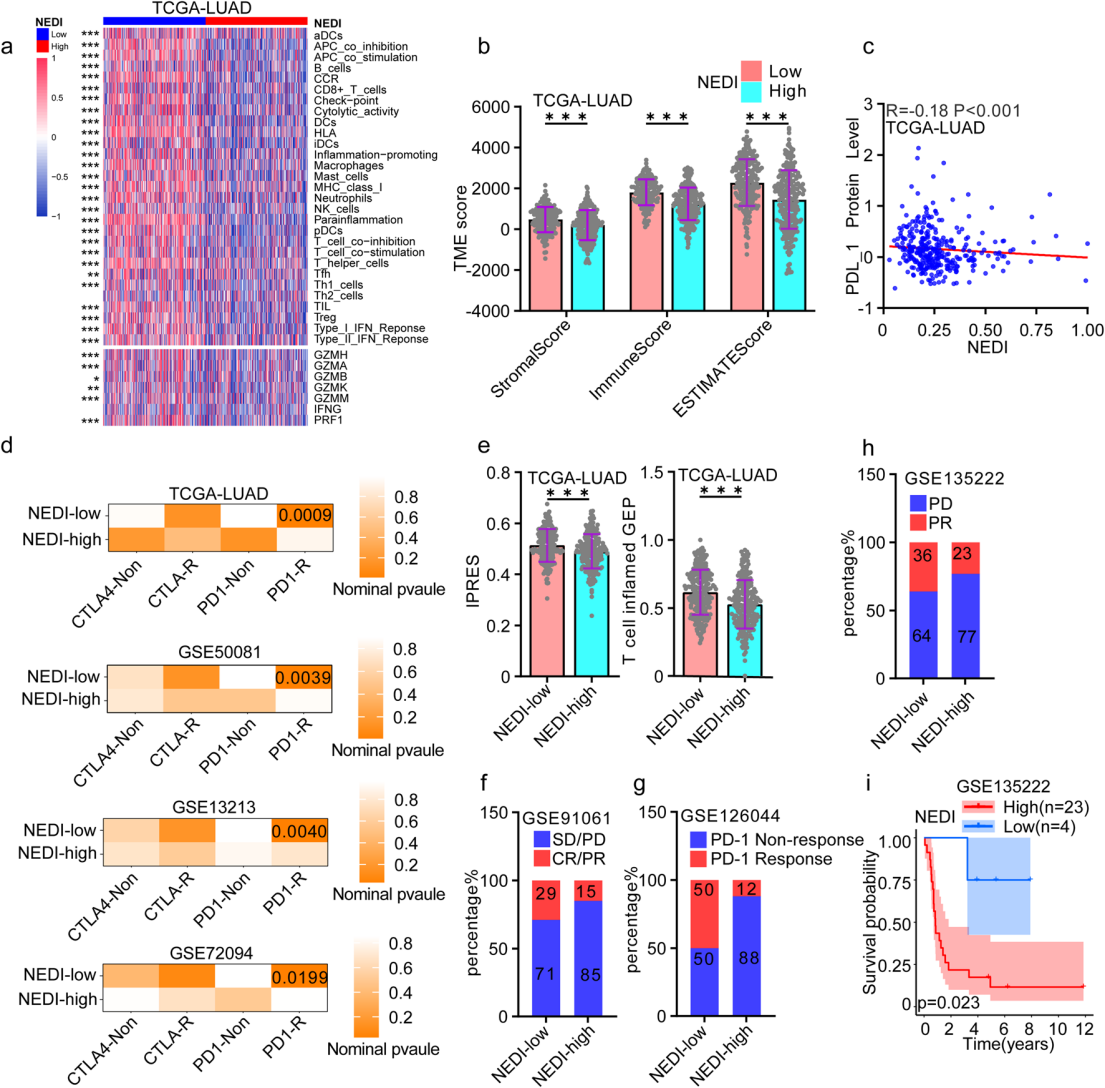
TME cell infiltration features and immunotherapy response in the NEDI-low and high cohorts. **a** Heatmap of 29 immune-related pathways scored by ssGSEA algorithm and immune effector molecules expression in NEDI-low and high subgroups in TCGA-LUAD. **b** Comparisons of stromal score, immune score and ESTIMATE score between the NEDI-low and high subgroups in TCGA-LUAD. Data were analysed by the student's t test. **c** Correlation analysis between the NEDI and PD-L1 protein levels in TCGA-LUAD. Data were analysed by Spearman correlation analysis. **d** Submap analysis demonstrated that the NEDI-low subgroup in LUAD cohorts (TCGA-LUAD, GSE13213, GSE50081 and GSE72094) were nearly identical to PD1-response (PD1-R) group defined in the melanoma cohort. **e** Comparisons of the expression of immune checkpoint blockade (ICB) response-related signatures (T-cell inflamed GEP and IPRES) between the NEDI-low and high subgroups in TCGA-LUAD. Data were analysed by Student's t test. **f**–**h** Clinical response to immunotherapy in NEDI-low and high subgroups in NSCLC patients (GSE91061, GSE126044 and GSE135222). **i** Kaplan‒Meier curve of progression-free survival (PFS) according to the NEDI values of NSCLC patients in the GSE135222 cohort. *P < 0.05, **P < 0.01, *** *P* < 0.001

To assess the relationships between NED and the tumour immune micro-environment, we analysed 29 immune-related pathways in the NEDI-low and NEDI-high subgroups by applying the ssGSEA algorithm. We observed that higher NEDI values were significantly associated with reduced immune cell infiltration and immune effector molecule expression in the TCGA-LUAD and GEO-LUAD cohorts (GSE13213, GSE50081 and GSE72094) (Fig. 5a and Figure S5a). Moreover, we found that the NEDI-low subgroup had higher stromal scores, immune scores and ESTIMATE scores calculated using the ESTIMATE algorithm (Fig. 5b and Figure S5b). These results showed that the NEDI-low subgroup was more likely to have an inflamed immune microenvironment. We also found an association of higher NEDI values with lower PD-L1 protein expression in the TCGA-LUAD dataset (Fig. 5c). We expected that tumours with low NED would be susceptible to immune checkpoint blockade (ICB) treatment because they have sufficient immune cell infiltration and preexisting upregulation of the PD-L1 pathway.

We further analysed the probability of ICB response in the NEDI-low and NEDI-high subgroups through SubMap analysis by analysing the NEDI in a published melanoma immunotherapy dataset [56].The results showed that the NEDI-low subgroup was likely to have a favourable response to anti-PD-1 therapy. It has been reported that the T-cell inflamed gene expression profile (GEP) and innate anti-PD-1 resistance gene signature (IPRES) can predict tumour response to ICB therapy [57, 58]. The T-cell inflamed GEP and IPRES were enriched in the NEDI-low subgroup in comparison to the NEDI-high subgroup (Fig. 5e and Figure S5c, d). We then assessed the difference in TCIA immunotherapy scores between the NEDI-low and NEDI-high subgroups and found that patients with lower NEDI values had higher TCIA immunotherapy scores in the TCGA-LUAD cohort (Figure S5e).

Finally, the value of the NEDI for predicting immunotherapy response was validated in three external NSCLC datasets (GSE91061, GSE126044 and GSE135222) with full clinical information about immunotherapy [29, 31, 33]. We found that NSCLC patients with low NEDI values were more likely to have considerable therapeutic benefits and clinical response to anti-PD-1/L1 immunotherapy than with those with high NEDI values (Fig. 5f–h). In particular, patients with low NEDI values exhibited longer progression-free survival (PFS) (Fig. 5i). These results indicated that tumours with low NED are more likely to respond to immunotherapy than those with high NED and that the NEDI can be used to predict the clinical response of LUAD to immunotherapy.

### The NEDI can serve as a predictive indicator of resistance to distinct chemotherapeutic agents

**Fig. 6:**
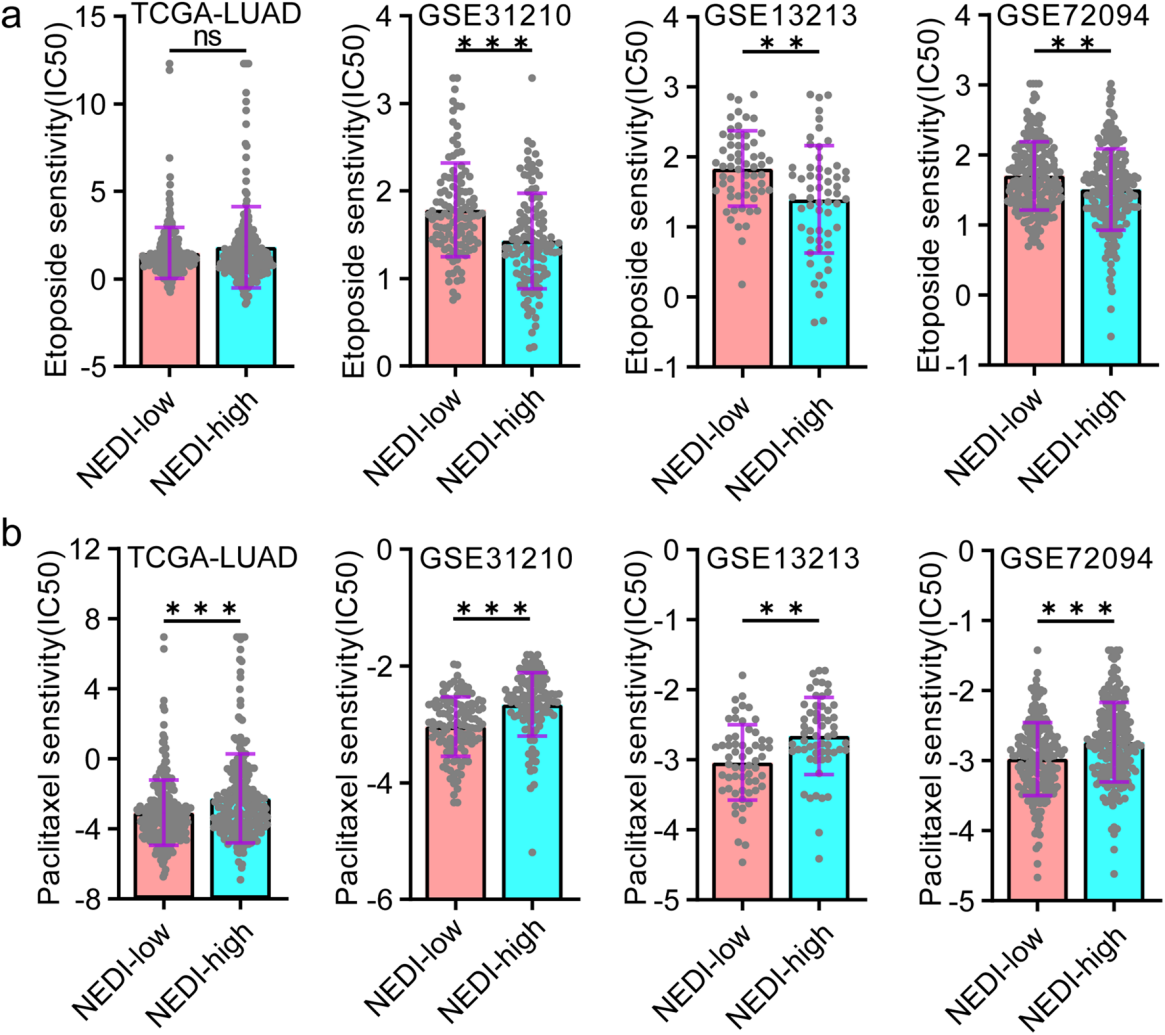
NEDI as a potential indicator to predict chemotherapy resistance. **a**, **b** Comparisons of molecular drug sensitivity between the NEDI-low and high subgroups based on IC50 values of etoposide and paclitaxel in TCGA-LUAD, GSE31210, GSE13213 and GSE72094 cohorts by applying “pRRophetic” algorithm. Data were analysed by the Mann–Whitney U test. *n.s.* no significance; *P < 0.05, **P < 0.01, *** *P* < 0.001

Chemotherapy is one of the standard treatments for LUAD patients. To further determine the value of the NEDI to guide treatment for LUAD, we used the “pRRophetic” algorithm to predict the response of LUAD samples to chemotherapeutic agents in multiple LUAD datasets (TCGA-LUAD, GSE31210, GSE13213 and GSE72094). We observed that LUAD samples with higher NEDI values displayed more sensitivity to etoposide, one of the most active agents for SCLC, but less sensitivity to paclitaxel than LUAD samples with lower NEDI values (Fig. 6a, b). Furthermore, 39 LUAD patients (GSE42127) who received adjuvant chemotherapy (mainly taxanes plus carboplatin) were analysed [32], and we observed that patients with higher NEDI values exhibited much worse prognoses (Figure S6). These results implied that paclitaxel-based regimens might not be effective in LUAD patients with high NEDI values and that etoposide-based comprehensive therapy would be a better alternative.

In summary, these results suggested that the NEDI can serve as a predictive indicator of resistance to distinct chemotherapeutic agents and that etoposide-based chemotherapy might be more effective in the treatment of LUAD with a high level of NED. However, clinical trials should be further conducted.

### Development and validation of a predictive nomogram based on the NEDI for LUAD

**Fig. 7:**
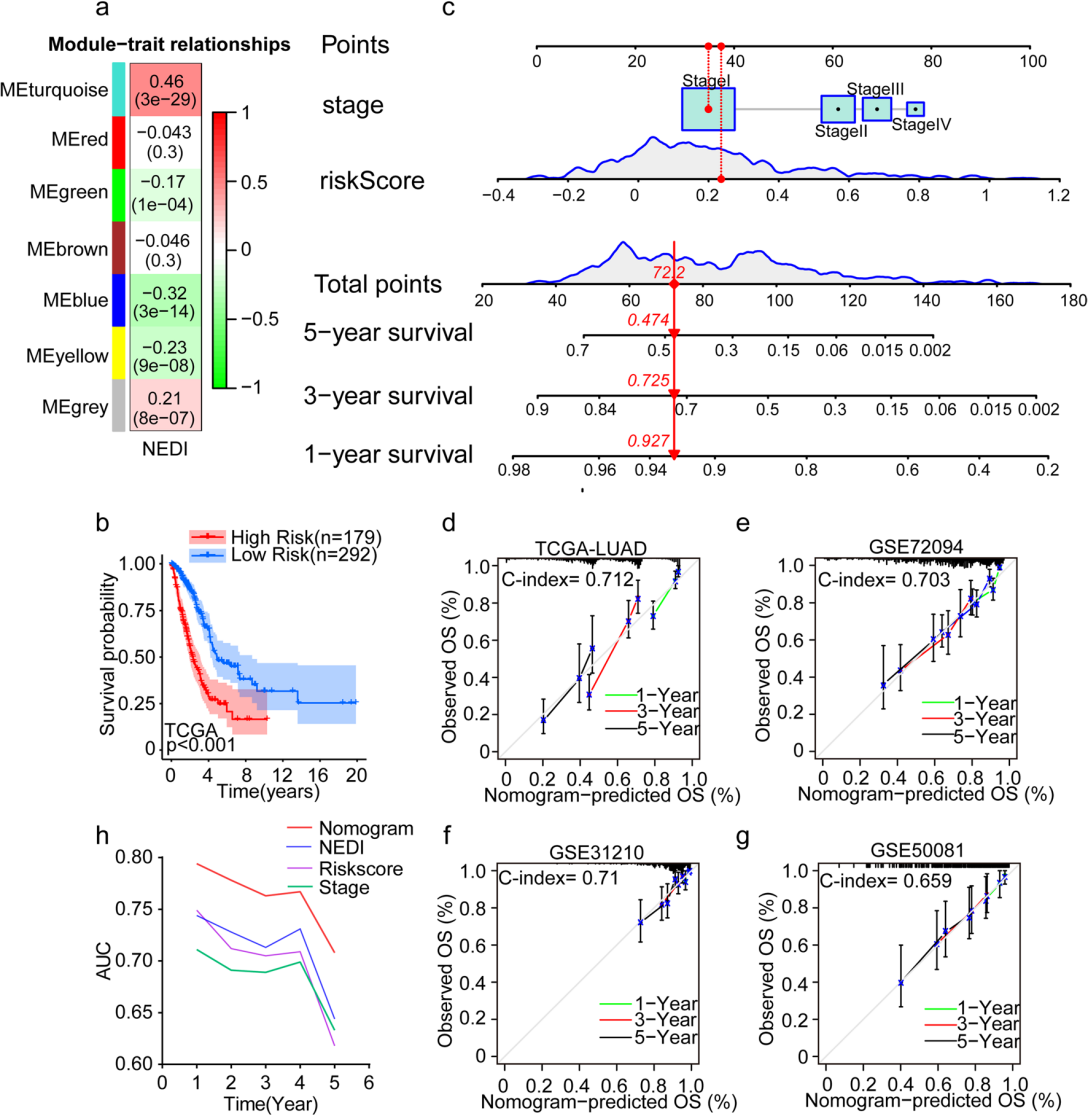
Establishing a prognostic model based on the NEDI for LUAD. **a** WGCNA analysis of key modules associated with the NEDI in TCGA-LUAD cohort. **b** Kaplan‒Meier curves of overall survival (OS) according to the risk score of patients in TCGA-LUAD cohort. **c** Prognostic nomogram of the stage and risk score in TCGA-LUAD cohort to predict overall survival. **d**–**g** Calibration curves of 1-,3-, 5-year OS prediction for the nomogram model in TCGA-LUAD, GSE72094, GSE31210, and GSE50081 cohorts. The 45-degree dotted line represents a perfect prediction, and the solid lines reflect the predictive performance of the nomogram. **h** Time‐dependent ROC curves of the nomogram, NEDI, risk score and clinical stage

To construct the NEDI-based prognostic model, 2693 mRNAs with a median absolute deviation (MAD) > 0.5 within the list of common genes were applied to perform WGCNA in the TCGA-LUAD cohort to find the key modules associated with the NEDI. The thresholding power of *β* = 5 (scale-free R^2^ = 0.9) was selected to construct a scale-free network (Figure S7a-c). Of the 7 modules identified, the turquoise module showed the highest positive correlation with the NEDI (Fig. 7a). Herein, 774 genes within the turquoise module were used to perform univariate Cox regression analysis, resulting in the identification of 385 survival-related genes. Next, those genes were selected for the Lasso Cox regression analysis, a recommended dimensionality-reduction method for the regression of high-dimensional data [59]. Finally, a NEDI-based risk model was established by calculating risk scores on the basis of the expression of 9 pivotal genes (CXCL17, DKK1, KRT8, IRX5, ENPP5, RHOV, MELTF, ESYT3, PLEK2) and respective coefficients for each patient (Table S5). Using the optimal cut-off points as the threshold, patients in the TCGA-LUAD cohort were divided into low- and high-risk subgroups, and the Kaplan‒Meier survival curves showed that patients with lower risk scores had better clinical outcomes (Fig. 7b). Moreover, the prognostic value was validated in GEO-LUAD datasets, including GSE72094, GSE31210 and GSE50081 (S7d-f). In addition, risk score and clinical stage were identified as prognostic factors after univariable Cox regression analysis and were used to construct a comprehensive nomogram, which can provide clinicians with the prognosis of patients based on their total points (Fig. 7c and Figure S7g). The calibration curves of 1- 3 and 5-year OS prediction were close to the ideal performance in both the training (TCGA-LUAD, C-index = 0.712) and external validation cohorts (GSE72094, C-index = 0.703, and GSE31210, C-index = 0.71 and GES50081, C-index = 0.659) (Fig. 7d–g). In addition, time-dependent ROC analysis demonstrated that the nomogram exhibited a much more powerful capacity for survival prediction than the NEDI and clinical stage, with an average AUC above 0.75 during a follow-up of 4 years (Fig. 7h). In conclusion, our results imply that the NEDI-based prognostic model can guide clinical treatment and predict prognosis to a certain extent in patients with LUAD.

## Discussion

Neuroendocrine features can be found in 10–30% of NSCLC tumours with no evidence of neuroendocrine transformation histologically, and these tumours are collectively referred to as NSCLC with NED [5]. In contrast to conventional histological classifications, NSCLC-NED can be identified by IHC or ultrastructural pathology examination rather than light microscopy [6, 7]. Distinct studies have been carried out to explore the treatment response and prognosis of NSCLC-NED, but the results are contradictory. Some of these contradictory findings may be particularly related to the different stages of disease, treatments received and antibodies used in IHC [53, 60, 61]. Despite these advances, little is known about NSCLC-NED therapy, which leads to uncertainty about appropriate treatments and prognostic implications for clinicians.

Gene expression data obtained from tumour biopsy samples reflect the overall state of multiple cell subpopulations in a sample. Traditional methods based on nonnegative matrix factorization or other matrix decomposition techniques for collecting gene expression data have difficulty identifying all tumour cell subtypes in a single optimization [12]. The OCLR machine learning algorithm, which is a novel one-class method based on logistic regression, has been demonstrated to have an advantage over its binary predictor counterparts in identifying specific cell types in heterogeneous cell populations [13]. In the present study, we chose the one-class framework because it is robust in the absence of a “negative” class. The NED index (NEDI) to quantify the degree of NED in NSCLC was established by the OCLR algorithm trained on 50 SCLC cell lines with the expression patterns of 13,279 common genes. Our results showed that the NEDI was positively associated with the level of neuroendocrine features, and this model was validated in different independent lung cancer cohorts. Application of the NEDI revealed a high degree of intratumor heterogeneity with respect to neuroendocrine features and provided insight into quantifying the degree of NED in NSCLC. To the best of our knowledge, this is the first study to develop a classification tool and machine-learning algorithm for the analysis of a spectrum of neuroendocrine features in NSCLC based on transcriptome data.

In this study, we initially analysed the relationship between the NEDI and molecular and clinical features and found a strong association between the NEDI and LUAD.2, one of the molecular subtypes of LUAD previously defined by TCGA and known to exhibit higher tumour ploidy and mutation rates and to frequently harbour TP53 mutations [50]. We found that a high NEDI was strongly correlated with somatic mutations in the TP53 and RB1 genes and tobacco carcinogens, which have been reported to be associated with the development of SCLC, the most common form of neuroendocrine lung cancer. These results further confirmed that the NEDI could be used to quantitatively determine the degree of NED in NSCLC.

Different mutational and immune landscapes were also observed between groups with different levels of NED (categorized based on the NEDI). We found that LUAD samples with higher NEDI values showed more genomic variants, and there was a strong association between the NEDI and TMB. It has been shown that patients with tumours with a high TMB are likely to benefit from immunotherapy [62]. Unexpectedly, a higher NEDI was associated with reduced immune cell infiltration and lower PD-L1 expression in LUAD despite the high TMB in our results. The therapeutic benefits and clinical response to anti-PD-1/L1 immunotherapy in LUAD patients with different NEDI values were predicted by calculating the T-cell inflamed GEP and IPRES and performing SubMap analysis. Moreover, the model was validated in three independent datasets of lung cancer immunotherapy. Our results showed that LUAD patients with high NEDI values were less likely to respond to ICB treatments. Our findings indicated that the NEDI might help predict the efficacy of immunotherapy and that TMB might not be a fully reliable biomarker for predicting the response to immunotherapy in LUAD patients.

Furthermore, we performed GSEA to investigate the biological processes associated with NED in LUAD and found that cell-cycle-related cancer hallmark gene sets significantly contributed to the positive side and immune-related cancer hallmark gene sets were significantly enriched on the negative side of the NED signature. This further confirmed that sufficient immune cell infiltration might exist within LUAD samples with low degrees of NED and that those patients might benefit from immunotherapy. Moreover, CMap analysis using the gene expression signatures of tumour samples with different NEDI values was performed and showed that tubulin inhibitors were most likely to be effective based on their MoA, indicating that microtubule-based functions, such as cell migration, cell division, and intracellular trafficking, might be involved in NED. In addition, we found that tumours with strong potential for NED generally had more rapid cell proliferation and reduced angiogenesis within microenvironments in LUAD, suggesting that LUAD samples with high NEDI values might be more susceptible to chemotherapy and less sensitive to antiangiogenic agents, which might result in better prognoses in patients with LUAD with high degrees of NED.

Neuroendocrine features arise from progenitor cells that accumulate mutations in oncogenic pathways or from differentiation of non-neuroendocrine cancer cells into cancer cells with neuroendocrine features via alteration of core signalling pathways that regulate the neuroendocrine phenotypes in NSCLC. In this study, we observed that primary LUAD tumour samples exhibited neuroendocrine features that were similar to those of nontumour samples obtained from matched adjacent normal tissues of origin, suggesting that the cancer cells had inherent neuroendocrine features rather than the features developing because of differentiation induced by the tumour microenvironment in NSCLC. However, further validation using other genomic profiles and laboratory experiments is required to validate the findings.

Stem-cell-like features are defined as the potential for self-renewal and differentiation in a cell of origin that possesses the ability to give rise to all cell types in an adult organism [63]. In our results, we found that EGFR-TKI-resistant samples had a higher NEDI than TKI-sensitive samples from patients with LUAD with EGFR mutations. Moreover, the NEDI was positively correlated with stemness features in LUAD. These results suggest that LUAD samples with high levels of neuroendocrine features exhibit progenitor and stem-cell-like features and high potential for histological transformation into SCLC, which may result in acquired resistance to EGFR-TKIs.

More interestingly, we observed that LUAD samples with higher NEDI values displayed more sensitivity to etoposide, one of the most active agents used for small-cell lung cancer therapy, but less sensitivity to paclitaxel (microtubule-targeting agents) by using the “pRRophetic” algorithm. Consistently, a much worse prognosis in the higher NEDI subgroup was validated in LUAD patients who received adjuvant chemotherapy with a taxane. This may be partly explained by the dedifferentiation of neuroendocrine cells induced by microtubule-targeting agents, which results in reduced cell cycle progression and less sensitivity to chemotherapeutic agents. It also indicated that paclitaxel-based regimens might not be effective in LUAD patients with high NEDI values and that etoposide-based combination therapy would be a better alternative. Chemotherapy has been introduced into the standard treatment for LUAD patients with acquired resistance to EGFR-TKIs [3]. Hence, etoposide-based regimens might be more effective in LUAD patients with acquired resistance to EGFR-TKIs.

Different sensitivities and specificities of the agents used in IHC analysis may lead to significant variation in reported incidence and underly the contradictory results about the prognosis of NSCLC-NED [60]. An integrative analysis based on the transcriptome would provide a reliable estimation of markers for cancer with advantages of being amenable to standardization and avoiding interpretation bias [10, 11]. Hence, the mRNA-based NEDI model would be more reliable for the evaluation of NED in NSCLC than IHC analysis. However, different transcriptome analysis platforms utilize various equipment types and physical principles for detecting output signals, as well as different library preparation protocols, leading to substantial batch effects and incompatibility problems [64, 65]. Moreover, contradictory mRNA expression results have been obtained from the use of various sources of clinical biomaterials; for example, high-integrity RNAs may be obtained from fresh tissue specimens rather than formalin-fixed paraffin-embedded tissue samples [66]. Hence, the mRNA-based NEDI model has some limitations in clinical utility and thus should be validated and modified in subsequent studies.

We found that LUAD patients with a higher NEDI exhibited a better prognosis in most LUAD datasets, indicating that the NEDI (calculated based on transcriptomic data) exhibited high prognostic value for LUAD. By applying the NEDI model, a prognostic NED-related signature was established and tested in the TCGA-LUAD cohort. A NEDI-based risk model was established by calculating risk scores on the basis of the expression of 9 pivotal genes. We found that patients with lower risk scores showed better clinical outcomes. In addition, the risk score and clinical stage exhibited powerful prognostic capacity and were incorporated into a comprehensive nomogram that achieved accurate survival prediction.

Although this study achieved comprehensive investigation of the mutational landscapes, altered pathways and immune characteristics of subgroups with different NEDI values and suggested potential personalized treatments for patients with LUAD based on the NEDI model, future studies including evaluations of molecular mechanisms and clinical trials should be conducted to validate our findings and ensure the clinical utility of the NEDI as a biomarker. These issues are worthy of further study.

## Conclusion

Collectively, our findings shed some light on the field of NED and we developed a new index based on the OCLR algorithm to quantitatively evaluate neuroendocrine features in LUAD. Our study provides a strategy for applying NEDI-based risk stratification to guide decision-making regarding immunotherapy and chemotherapy.

## Supplementary Information

Supplementary file1

Supplementary file2

Supplementary file3

Supplementary file4

Supplementary file5

Supplementary file6

Supplementary file7

Supplementary file8

Supplementary file9

Supplementary file10

Supplementary file11

Supplementary file12

## Data Availability

All data generated or analysed during this study are included in this published article and its supplementary information files. Lung cancer transcriptomics datasets analyzed in this research can be found at: The Cancer Genome Atlas (TCGA) (https://portal.gdc.cancer.gov/), Clinical Proteomic Tumor Analysis Consortium (CPTAC) (https://www.cbioportal.org), Cancer Cell Line Encyclopedia (CCLE) (http://www.broadinstitute.org/ccle), and Gene Expression Omnibus (GEO) (https://www.ncbi.nlm.nih.gov/geo).

## Abbreviations

NSCLC: Non-small cell lung cancer
OCLR: One-class logistic regression
NED: Neuroendocrine differentiation
NEDI: Neuroendocrine differentiation index
SCLC: Small cell lung cancer
LUAD: Lung adenocarcinoma
LUSC: Lung squamous cell carcinoma
EGFR-TKIs: Epidermal growth factor receptor -tyrosine kinase inhibitors
NSCLC-NED: Non-small cell carcinomas with neuroendocrine differentiation
IHC: Immunohistochemistry
CgA: Chromogranin A
CD56: Neural cell adhesion molecule 1
Syp: Synaptophysin
NSE: Neuron-specific enolase
TCGA: The Cancer Genome Atlas
CPTAC: Clinical Proteomic Tumor Analysis Consortium
CCLE: Cancer Cell Line Encyclopedia
GEO: Gene Expression Omnibus
BSA: Bovine serum albumin
MAF: Mutation Annotation Format
TME: Tumor microenvironment
TMB: Tumour mutation burden
ssGSEA: Single-sample gene-set enrichment analysis
IPS: Immunophenoscore
T-cell inflamed GEP: T-cell inflamed gene expression profile
IPRES: Innate anti-PD-1 resistance
Submap: Subclass mapping
CMap: Connectivity Map
LCNC: Large-cell neuroendocrine carcinomas
CC: Carcinoids
SNPs: Single-nucleotide polymorphisms
SNVs: Single-nucleotide variants
INSs: Insertions
DELs: Deletions
DEGs: Differentially expressed genes
EMT: Epithelial mesenchymal transition

## Code availability

The statistics and analysis of data were based on open source software programming (R project, https://www.r-project.org/) and the information about key software and algorithms used in this study are provided in Table S3.
