# Supplementary figures and images for: Depiction of neuroendocrine features associated with immunotherapy response using a novel one-class predictor in lung adenocarcinoma
Source: Discov Oncol. 2023 May 18;14:71. doi: 10.1007/s12672-023-00693-4 (PMC10195954; doi:10.1007/s12672-023-00693-4)

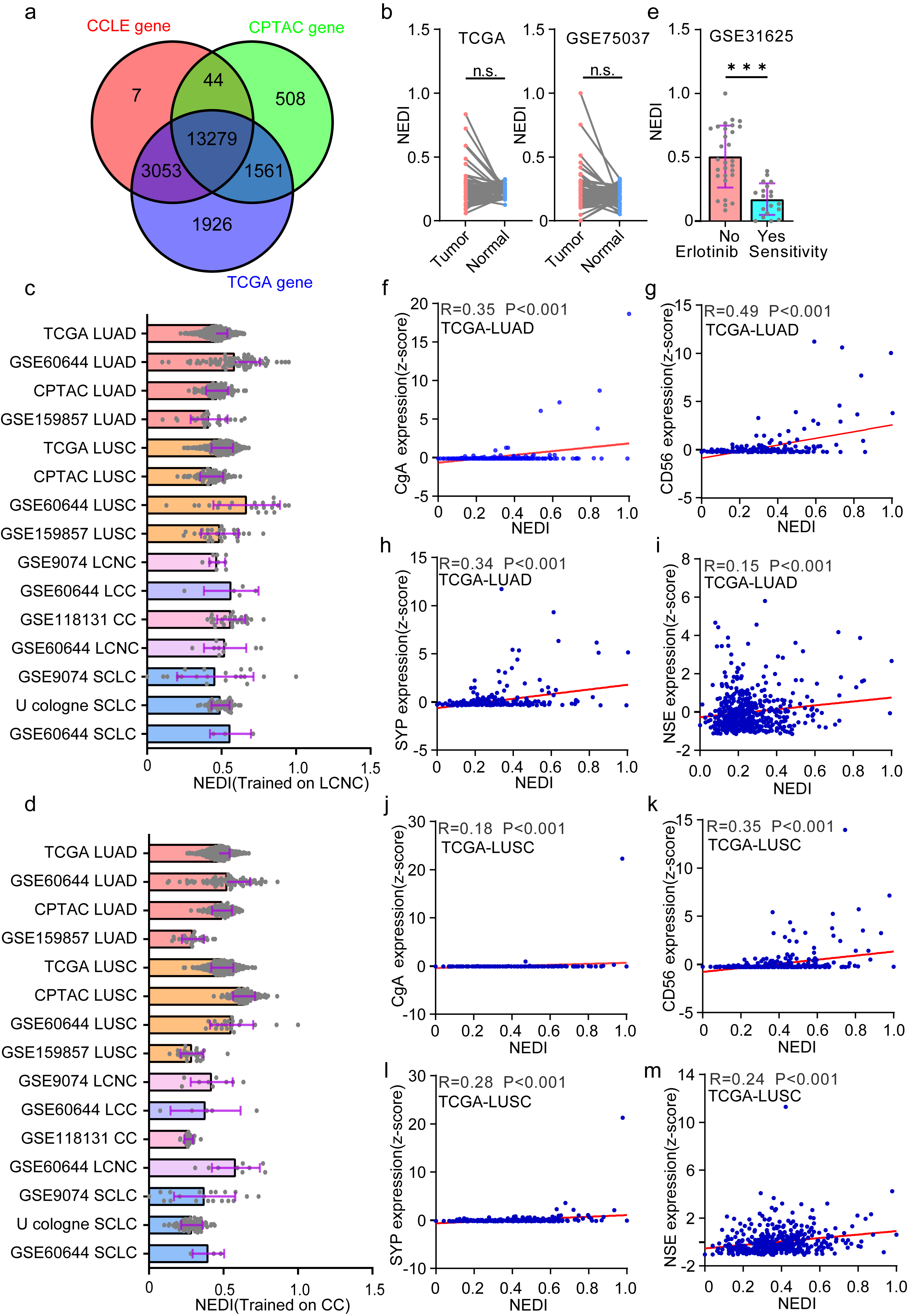

Supplement: Supplementary file 1 — Supplementary file1 [file 12672_2023_693_MOESM1_ESM.tif]

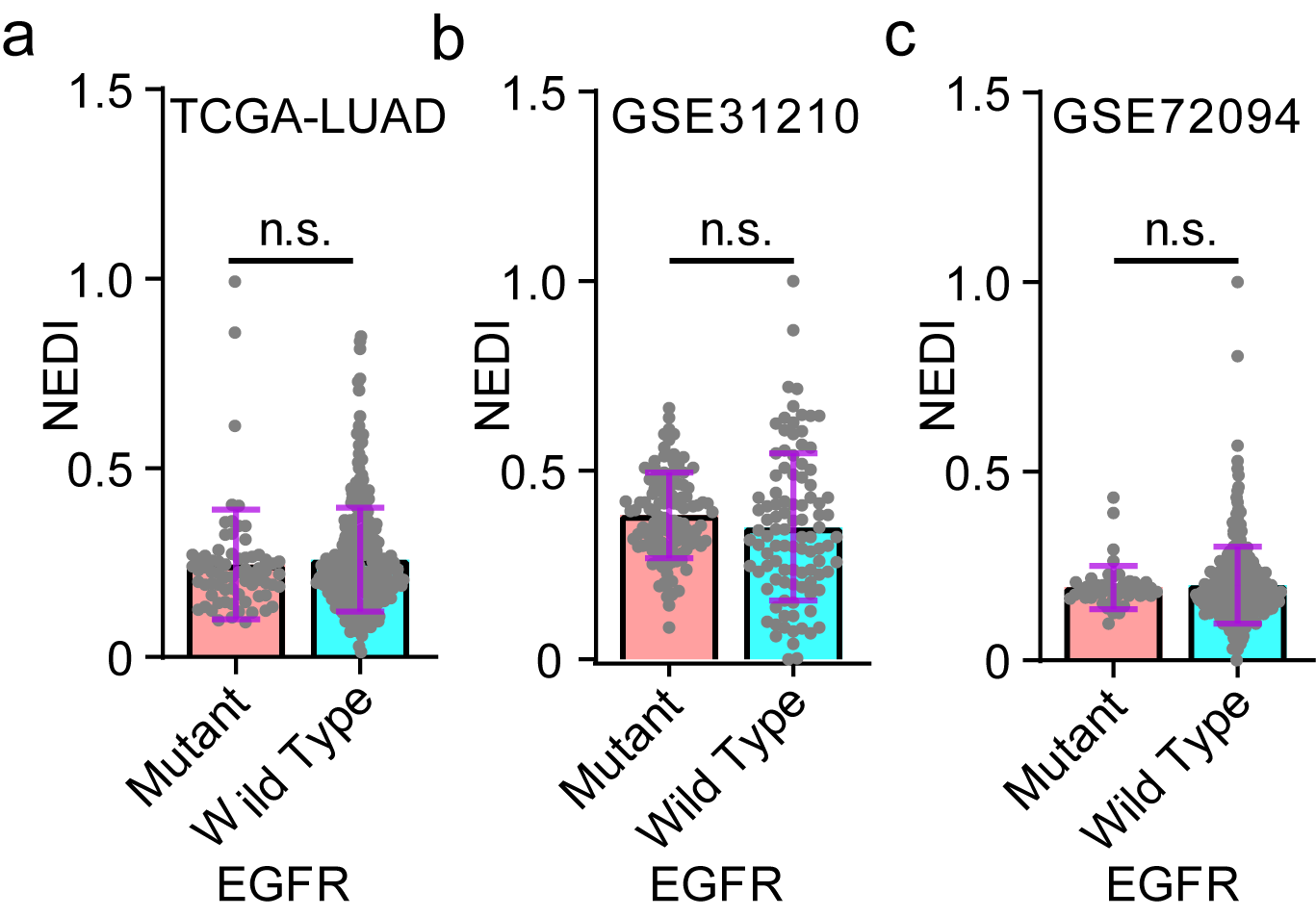

Supplement: Supplementary file 2 — Supplementary file2 [file 12672_2023_693_MOESM2_ESM.tif]

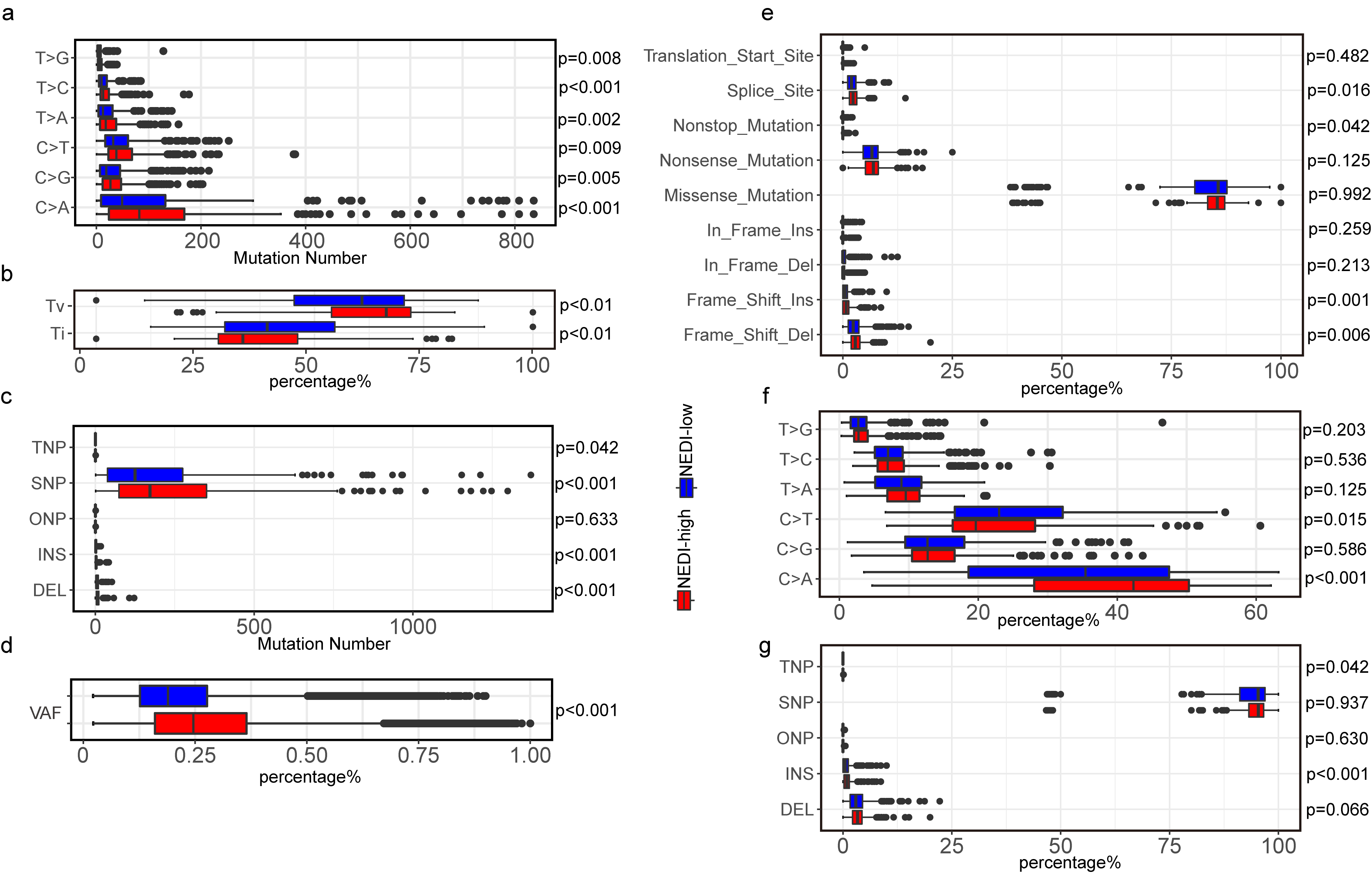

Supplement: Supplementary file 3 — Supplementary file3 [file 12672_2023_693_MOESM3_ESM.tif]

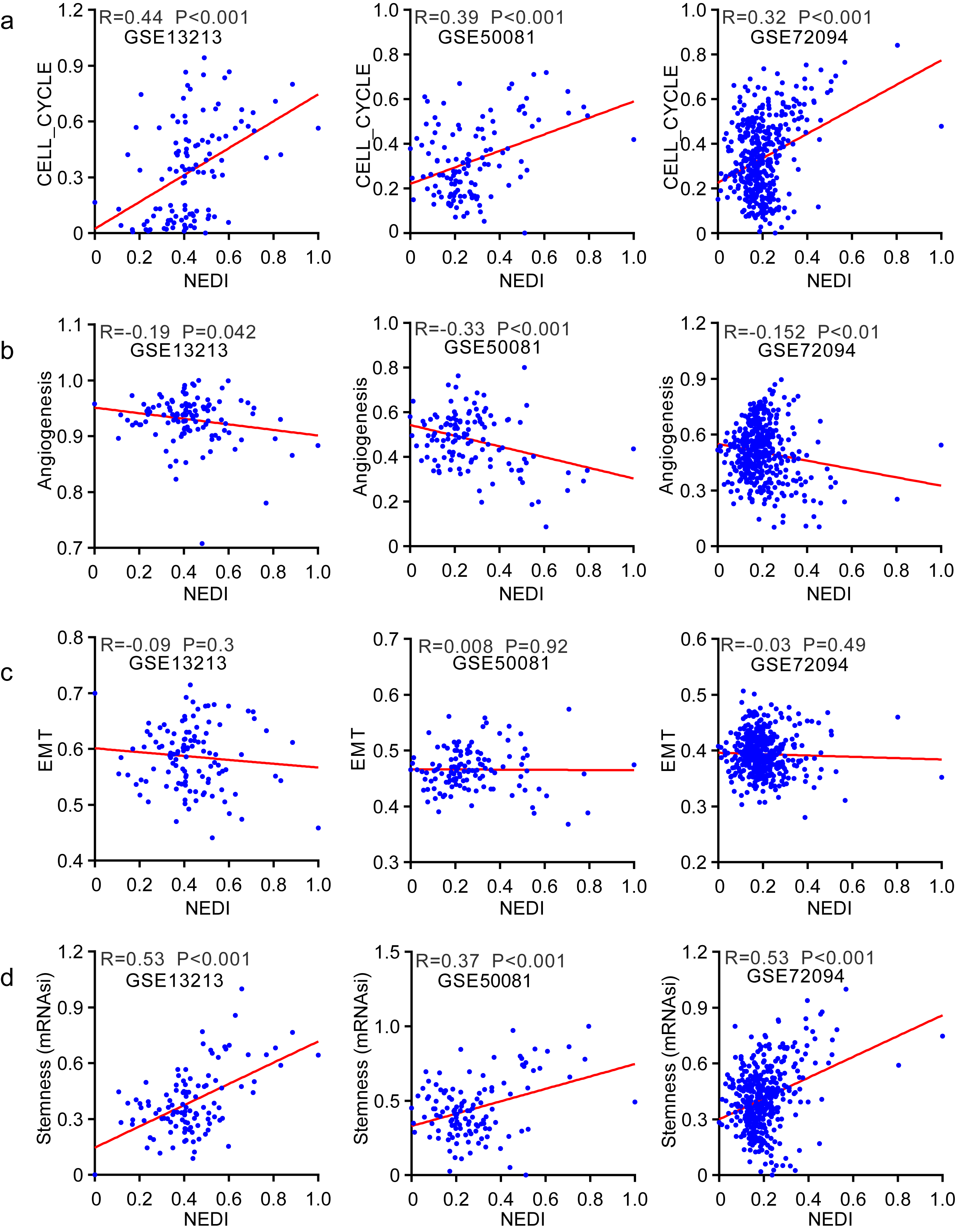

Supplement: Supplementary file 4 — Supplementary file4 [file 12672_2023_693_MOESM4_ESM.tif]

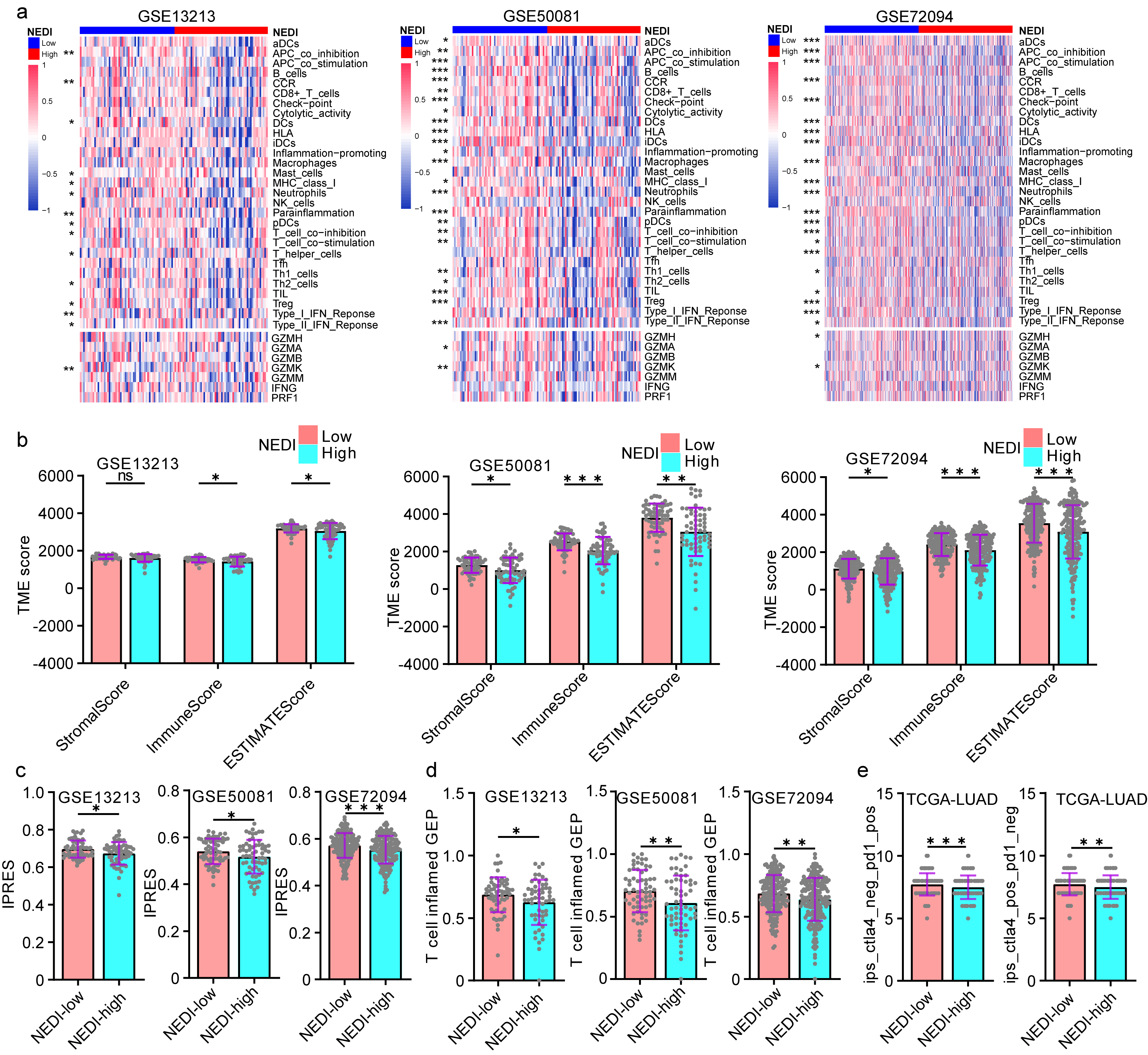

Supplement: Supplementary file 5 — Supplementary file5 [file 12672_2023_693_MOESM5_ESM.tif]

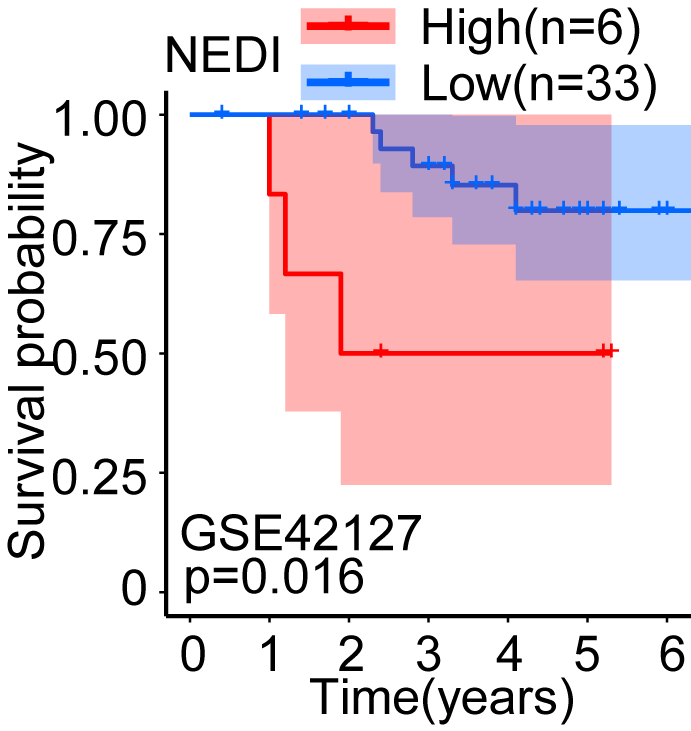

Supplement: Supplementary file 6 — Supplementary file6 [file 12672_2023_693_MOESM6_ESM.tif]

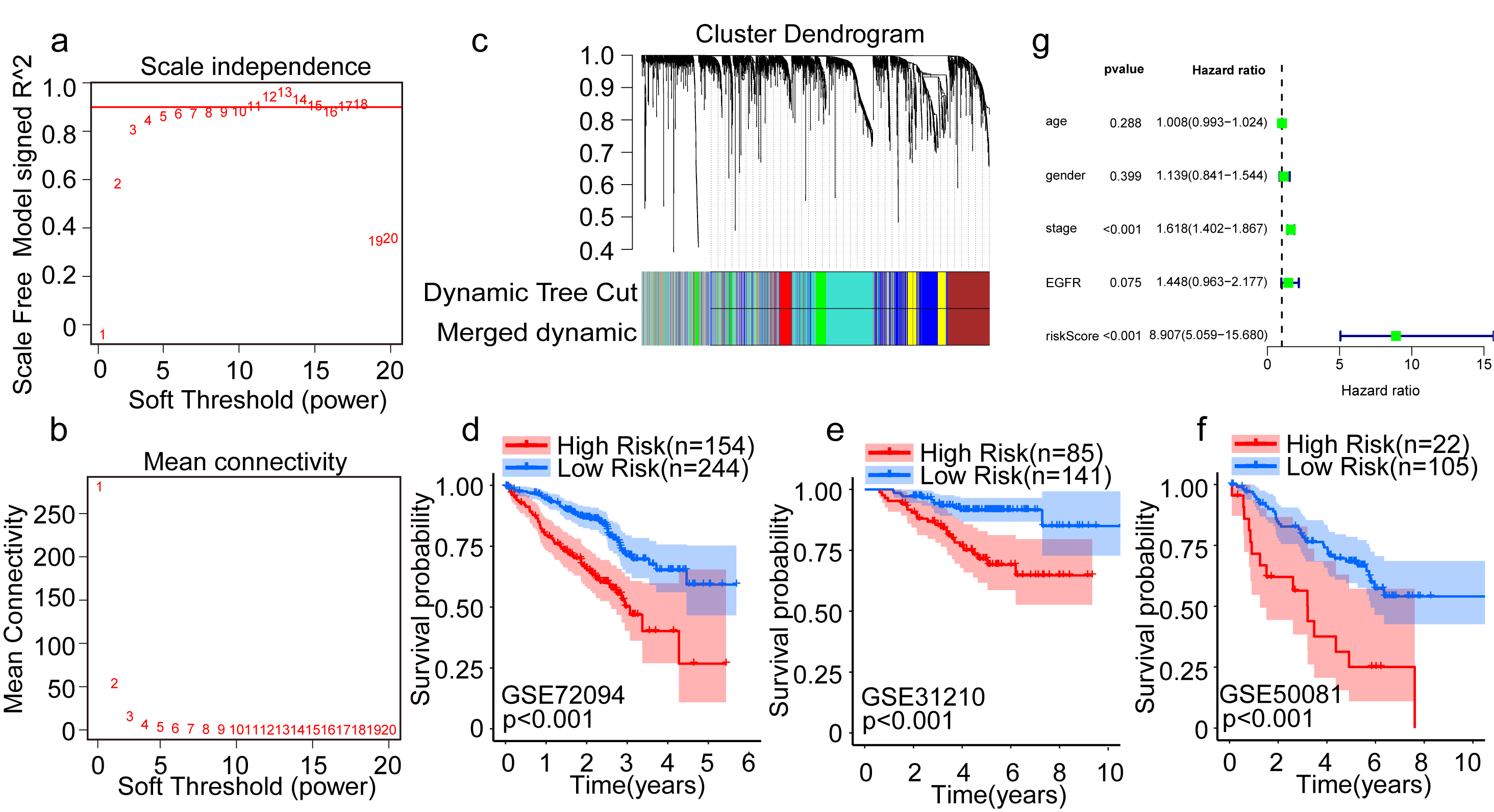

Supplement: Supplementary file 7 — Supplementary file7 [file 12672_2023_693_MOESM7_ESM.tif]
