# Supplementary material for: Depiction of neuroendocrine features associated with immunotherapy response using a novel one-class predictor in lung adenocarcinoma
Source: Discov Oncol. 2023 May 18;14:71. doi: 10.1007/s12672-023-00693-4 (PMC10195954; doi:10.1007/s12672-023-00693-4)
Supplement: Supplementary file 8 — Supplementary file8 [file 12672_2023_693_MOESM8_ESM.docx]

Table S1. Basic information of lung cancer cohorts used in this study

| Cohort Names | Platform used | Histological type | Number of input | Source |
| --- | --- | --- | --- | --- |
| CCLE lung cancer cell lines | Affymetrix U133 2.0 | SCLC, NSCLC | 185 | Mahmoud Ghandi *etal.* Nature.2019May;569(7757):503-508 |
| TCGA-LUAD | Illumina HumanHT-12 V4.0 | LUAD | 598 | NIH Genomic Data Commons (GDC) |
| TCGA-LUSC | Illumina HumanHT-12 V4.0 | LUSC | 502 | NIH Genomic Data Commons (GDC) |
| CPTAC-LUAD | Illumina | LUAD | 110 | Michael A Gillette *et al*. Cell. 2020 Jul 9;182(1):200-225.e35 |
| CPTAC-LUSC | Illumina | LUSC | 108 | Shankha Satpathy *et al.* Cell. 2021 Aug 5;184(16):4348-4371.e40. |
| U cologne SCLC | Affymetrix 6.0 | SCLC | 120 | Julie George *et al.* Nature. 2015 Aug 6;524(7563):47-53. |
| GSE159857 | GPL18573 Illumina NextSeq 500 | LUAD, LUSC | 58 | Jose Thaiparambil *et al*. Cancer Med.2022 Jun 8 |
| GSE60644 | GPL10558 Illumina HumanHT-12 V4.0 expression beadchip | LUAD, LCC, SCLC, LCNC | 117 | Anna Karlsson *et al.* Clin Cancer Res. 2014 Dec 1;20(23):6127-40. |
| GSE9074 | GPL570Affymetrix Human Genome U133 Plus 2.0 Array | SCLC, LCNC | 29 | Kano M *et al.* |
| GSE118131 | GPL11154 Illumina HiSeq 2000 | CC | 30 | Saurabh V Laddha *et al*. Cancer Res. 2019 Sep 1;79(17):4339-4347. |
| GSE64322 | GPL570 Affymetrix Human Genome U133 Plus 2.0 Array | NSCLC | 10 | Matthew J Niederst *et al.* Nat Commun. 2015 Mar 11;6:6377. |
| GSE1037 | GPL962 CHUGAI 41K | SCLC, LCNC, LCC, LUAD | 72 | Takeshi Fujiwara *et al.* Lung Cancer. 2012 Jan;75(1):119-25. |
| GSE31625 | GPL96 Affymetrix Human Genome U133A Array | LUAD | 48 | Justin M Balko *et al.* BMC Genomics. 2006 Nov 10;7:289. |
| GSE31210 | GPL570 Affymetrix Human Genome U133 Plus 2.0 Array | LUAD | 226 | Mai Yamauchi *et al.* PLoS One. 2012;7(9):e43923. |
| GSE50081 | GPL570 Affymetrix Human Genome U133 Plus 2.0 Array | LUAD | 127 | Sandy D Der *et al.* J Thorac Oncol. 2014 Jan;9(1):59-64. |
| GSE13213 | GPL6480 Agilent-014850 Whole Human Genome Microarray 4x44K G4112F (Probe Name version) | LUAD | 117 | Shuta Tomida *et al.* J Clin Oncol. 2009 Jun 10;27(17):2793-9. |
| GSE72094 | GPL15048 Rosetta/Merck Human RSTA Custom Affymetrix 2.0 microarray | LUAD | 442 | M B Schabath *et al.* Oncogene. 2016 Jun 16;35(24):3209-16. |
| GSE91061 | GPL9052 Illumina Genome Analyzer | NSCLC | 109 | Nadeem Riaz *et al.* Cell. 2017 Nov 2;171(4):934-949.e16. |
| GSE126044 | GPL16791 Illumina HiSeq 2500 | NSCLC | 16 | Jae-Won Cho *et al.* Exp Mol Med. 2020 Sep;52(9):1550-1563. |
| GSE42127 | GPL6884 Illumina HumanWG-6 v3.0 expression beadchip | LUAD | 39 | Suzie K Hight *et al.* Neoplasia. 2020 Aug;22(8):294-310. |
| GSE75037 | GPL6884 Illumina HumanWG-6 v3.0 expression beadchip | LUAD | 166 | Luc Girard *et al.* Clin Cancer Res. 2016 Oct 1;22(19):4880-4889. |
| GSE135222 | GPL16791 Illumina HiSeq 2500 | NSCLC | 27 | Jung H *et al.* Nat Commun. 2019 Sep 19;10(1):4278. |

NSCLC, non-small cell lung cancer; SCLC, small cell lung cancer; LUAD, lung adenocarcinoma; LUSC, lung squamous cell carcinoma; LCNC, large cell neuroendocrine carcinoma; LCC, large cell carcinoma; CC, carcinoid
