# Supplementary material for: Depiction of neuroendocrine features associated with immunotherapy response using a novel one-class predictor in lung adenocarcinoma
Source: Discov Oncol. 2023 May 18;14:71. doi: 10.1007/s12672-023-00693-4 (PMC10195954; doi:10.1007/s12672-023-00693-4)
Supplement: Supplementary file 10 — Supplementary file10 [file 12672_2023_693_MOESM10_ESM.docx]

| Table S3. Key Software and Algorithms used in this study | | | |
| --- | --- | --- | --- |
| Software and Algorithms | Source | | Identifier |
| R 4.2.1 | R Development Core Team, 2022 | https://www.R-project.org | |
| Gelnet(v1.2.1) | Artem Sokolov *et al.* Pac Symp Biocomput. 2016;21:405-16. | https://cran.r-project.org/web/packages/gelnet/index.html | |
| Maftools(v2.8.05) | Anand Mayakonda *etal.* Genome Res. 2018 Nov;28(11):1747-1756. | https://bioconductor.org/packages/release/bioc/vignettes/maftools/inst/doc/maftools.html | |
| GSVA (v1.40.1) | Sonja Hänzelmann *etal*. BMC Bioinformatics. 2013 Jan 16;14:7. | https://www.bioconductor.org/packages/release/bioc/html/GSVA.html | |
| estimate (v1.0.13) | Kosuke Yoshihara *eta*l. Nat Commun. 2013;4:2612. | https://bioinformatics.mdanderson.org/estimate/rpackage.html | |
| fgsea(v1.24.0) | Sergushichev A *etal.* BioRxiv. 2016:060012 | https://bioconductor.org/packages/release/bioc/html/fgsea.html | |
| pRRophetic | Paul Geeleher *etal.* PLoS One. 2014 Sep 17;9(9):e107468. | https://osf.io/5xvsg/wiki/home/ | |
| Connectivity Map (CMap) | Justin Lamb *etal.* Science. 2006 Sep 29;313(5795):1929-35 | http://www.broad.mit.edu/cmap/ | |
| subclass mapping (Submap) | Michael Reich *etal.* Nat Genet. 2006 May;38(5):500-1. | https://cloud.genepattern.org/gp/pages/login.jsf | |
| WGCNA (v1.71) | Peter Langfelder *etal*. BMC Bioinformatics. 2008 Dec 29;9:559. | https://horvath.genetics.ucla.edu/html/CoexpressionNetwork/Rpackages/WGCNA/ | |
| Survival (v3.4-0) | Haiqun Lin&Daniel Zelterman *etal.* Technometrics Volume 44, 2002 - Issue 1 | http://cran.r-project.org/package=survival | |
| rms (v6.3-0) | Eduardo Núñez *etal* Review Rev Esp Cardiol. 2011 Jun;64(6):501-7. | https://cran.r-project -project.org/package=rms | |
| riskRegression(v 2022.11.28) | Thomas A Gerds *etal.* Review Biom J. 2008 Aug;50(4):457-79 | https://cran.r-project -project.org/package= riskRegression | |
| Pec (v2022.05.04) | Ulla B Mogensen *etal.* J Stat Softw. 2012 Sep;50(11):1-23. | https://cran.r-project -project.org/package=pec | |
| Limma(v3.48.3) | Matthew E Ritchie *etal.* Nucleic Acids Res. 2015 Apr 20;43(7):e47. | https://bioconductor.org/packages/release/bioc/html/limma.html | |
